# Autoimmunity Is a Significant Feature of Idiopathic Pulmonary Arterial Hypertension

**DOI:** 10.1164/rccm.202108-1919OC

**Published:** 2022-07-01

**Authors:** Rowena J. Jones, Eckart M. D. D. De Bie, Emily Groves, Kasia I. Zalewska, Emilia M. Swietlik, Carmen M. Treacy, Jennifer M. Martin, Gary Polwarth, Wei Li, Jingxu Guo, Helen E. Baxendale, Stephen Coleman, Natalia Savinykh, J. Gerry Coghlan, Paul A. Corris, Luke S. Howard, Martin K. Johnson, Colin Church, David G. Kiely, Allan Lawrie, James L. Lordan, Robert V. Mackenzie Ross, Joanna Pepke Zaba, Martin R. Wilkins, S. John Wort, Edoardo Fiorillo, Valeria Orru, Francesco Cucca, Christopher J. Rhodes, Stefan Graf, Nicholas W. Morrell, Eoin F. McKinney, Chris Wallace, Mark Toshner, UK National PAH study Consortium:, Marta Bleda, Charaka Hadinnapola, Matthias Haimel, Kate Auckland, Tobias Tilly, Jennifer M. Martin, Katherine Yates, Carmen M. Treacy, Margaret Day, Alan Greenhalgh, Debbie Shipley, Val Irvine, Fiona Kennedy, Shahin Moledina, Lynsay MacDonald, Eleni Tamvaki, Anabelle Barnes, Victoria Cookson, Latifa Chentouf, Souad Ali, Shokri Othman, Lavanya Ranganathan, J. Simon R. Gibbs, Gummadi Mahitha, Rosa DaCosta, Joy Pinguel, Natalie Dormand, Alice Parker, Della Stokes, Dipa Ghedia, Yvonne Tan, Tanaka Ngcozana, Ivy Wanjiku, Gary Polwarth, John Cannon, Karen K. Sheares, Dolores Taboda, Rob V. Mackenzie Ross, Jay Suntharalingam, Mark Grover, Ali Kirby, Iain Armstrong, Richard Trembath

**Affiliations:** Department of Medicine, University of Cambridge, Cambridge, UK; Freeman Hospital, Newcastle, UK; Golden Jubilee National Hospital, Glasgow, UK; Great Ormond Street Hospital, London, UK; Hammersmith Hospital, London, UK; National Heart & Lung Institute, Imperial College London; Royal Brompton Hospital, London; Royal Brompton Hospital, London, UK; Royal Free Hospital, London, UK; Royal Papworth Hospital, Cambridge, UK; Royal United Bath Hospitals, Bath, UK; Ali Hallamshire Hospital, Sheffield; Kings College London; 1 Heart and Lung Research Institute; 2 Royal Papworth Hospital; 3 Medical Research Council Biostatistics Unit; 4 Cambridge Biomedical Research Centre Phenotyping Hub, Department of Medicine, University of Cambridge; 5 Royal Free London National Health Service Foundation Trust, London, United Kingdom; 6 Freeman Hospital, Newcastle-upon-Tyne, United Kingdom; 7 Hammersmith Hospital, London, United Kingdom; 8 Scottish Pulmonary Vascular Unit, Golden Jubilee National Hospital, Glasgow, United Kingdom; 9 Sheffield Pulmonary Vascular Disease Unit, Royal Hallamshire Hospital, Sheffield, United Kingdom; 10 Department of Infection, Immunity and Cardiovascular Disease, University of Sheffield, Sheffield, United Kingdom; 11 Royal United Hospitals Bath National Health Service Foundation Trust, Bath, United Kingdom; 12 National Heart and Lung Institute, Faculty of Medicine, Imperial College London, London, United Kingdom; 13 Royal Brompton Hospital, London, United Kingdom; 14 Istituto di Ricerca Genetica e Biomedica, Consiglio Nazionale delle Ricerche, Cagliari, Italy; 15 University of Sassari, Sassari, Italy; 16 Cambridge Institute of Therapeutic Immunology and Infectious Disease, Jeffrey Cheah Biomedical Centre, Cambridge Biomedical Campus, Cambridge, United Kingdom; 17 Department of Medicine, University of Cambridge, Addenbrookes Hospital, Hills Road, Cambridge, United Kingdom

**Keywords:** autoimmune, pulmonary arterial hypertension, BMPR2, autoantibodies, IPAH

## Abstract

**Rationale:**

Autoimmunity is believed to play a role in idiopathic pulmonary arterial hypertension (IPAH). It is not clear whether this is causative or a bystander of disease and if it carries any prognostic or treatment significance.

**Objectives:**

To study autoimmunity in IPAH using a large cross-sectional cohort.

**Methods:**

Assessment of the circulating immune cell phenotype was undertaken using flow cytometry, and the profile of serum immunoglobulins was generated using a standardized multiplex array of 19 clinically validated autoantibodies in 473 cases and 946 control subjects. Additional glutathione S-transferase fusion array and ELISA data were used to identify a serum autoantibody to BMPR2 (bone morphogenetic protein receptor type 2). Clustering analyses and clinical correlations were used to determine associations between immunogenicity and clinical outcomes.

**Measurements and Main Results:**

Flow cytometric immune profiling demonstrates that IPAH is associated with an altered humoral immune response in addition to raised IgG3. Multiplexed autoantibodies were significantly raised in IPAH, and clustering demonstrated three distinct clusters: “high autoantibody,” “low autoantibody,” and a small “intermediate” cluster exhibiting high concentrations of ribonucleic protein complex. The high-autoantibody cluster had worse hemodynamics but improved survival. A small subset of patients demonstrated immunoglobulin reactivity to BMPR2.

**Conclusions:**

This study establishes aberrant immune regulation and presence of autoantibodies as key features in the profile of a significant proportion of patients with IPAH and is associated with clinical outcomes.

Pulmonary arterial hypertension (PAH) is characterized by severe remodeling of the pulmonary arteries causing increased pulmonary vascular resistance (PVR) and resulting in reduced cardiac output, right heart failure, and, despite the availability of numerous licensed therapies, reduced life expectancy (1). PAH is a mixed classification of pathologies, with a well-recognized female predominance, and has known associations with female-prevalent autoimmune diseases, including systemic sclerosis (SSc)-associated PAH, systemic lupus erythematosus (SLE), and Sjögren’s syndrome (2). The most common specialist center diagnosis remains idiopathic PAH (IPAH), in which the cause is still undetermined. Patients with IPAH show overlapping features of autoimmune disease, including inflammation and immune cell infiltration within the lung and putative circulating autoantibodies (1, 3–6). The concept that IPAH represents an undiagnosed autoimmune disease has circulated for decades (3, 7). The availability of whole-genome sequencing has clarified that only a modest proportion of patients with IPAH are likely to be reclassified as having rare disease-causing mutations, but adding weight to the autoimmune hypothesis, the major signal from common genetic variant analysis was located in an HLA-associated locus (8), and most recently, unsupervised clustering of whole-blood transcriptomics identified differences between immunoglobulin transcription as a determinant of good or poor survival among patients with IPAH (9). Irrespective of the causal versus associative nature of autoimmunity or inflammation, it remains an attractive target for drug repurposing. To date, no trial has successfully identified responders to immunomodulator therapies. A better understanding of the nature and proportion of patients with dysregulated immunity in IPAH is necessary to guide pathophysiology studies and future therapeutic trials.

Herein we describe the circulating immune phenotype and autoantibody profiling in PAH using patient samples collected as part of the UK National Cohort Study of Idiopathic and Heritable PAH. We demonstrate using a number of differing techniques that autoimmunity is associated with IPAH in the largest study to date in which autoimmunity clustering was associated with clinical phenotypes and outcomes. We also identify an uncommon putative autoantibody to the TGF-β (transforming growth factor-β) superfamily receptor, BMPR2 (bone morphogenetic protein receptor type 2), a key functional protein in PAH biology (10).

## Methods

### Subjects and Ethical Consent

All samples and data were obtained with written informed consent. Patients with diagnoses of heritable PAH (HPAH) or IPAH, pulmonary venoocclusive disease, or pulmonary capillary hemangiomatosis according to World Health Organization (WHO) classification and aged >18 years were recruited to the wider cohort study as previously described (8, 11).

For the immunophenotyping study, subjects were also recruited from the Pulmonary Vascular Diseases Unit at the Royal Papworth Hospital under the Papworth Hospital Tissue Bank (donation for the collection and storage of human biological material for research; Cambridgeshire East Research Ethics Committee reference 08/H0304/56, tissue bank project number T01990). Patients and control subjects for the autoantibody studies were recruited from the UK National Cohort Study of Idiopathic and Hereditary PAH (13/EE/0203; NCT 01907295), and additional control subjects were recruited from ProgeNIA (Genetics and Epidemiology of Aging-Associated Conditions; PROT 2171/CE) and An Integrated Approach to Dissect Determinants, Risk Factors and Pathways of Ageing of the Immune System (15/EE/0327, Integrated Research Application System identifier 188383). Serum samples were collected by the UK National Cohort Study of Idiopathic and Hereditary PAH between February 2014 and August 2018. Patients were censored in February 2021 using National Health Service records. Additional serum controls from healthy volunteers were used from the StratosPHere (Optimal Biomarkers to Ascertain Target Engagement in Therapies Targeting the BMPR2 Pathway in Pulmonary Arterial Hypertension [PAH]; 21/EE/0043) study. Serum samples were collected in serum-separating blood tubes, clotted for 30 minutes, and then centrifuged at 1,500 × *g* for 15 minutes; aliquots were stored at − 80° C until required.

### Peripheral Blood Mononuclear Cell Fraction Collection and Immunophenotyping

Peripheral blood mononuclear cell fractions were generated by density gradient centrifugation using Ficoll-Paque PLUS (GE Healthcare) from peripheral venous blood in 26 patients with IPAH and 29 healthy control subjects. Age and sex matching with healthy control subjects was undertaken on a 1:1 basis, with three patient samples excluded because of technically low-quality specific antibody staining. Immunophenotyping was performed using flow cytometry on fresh samples. Antibody staining panels and gating strategy were designed to detect populations of myeloid, B cells, and T cells on the basis of gating strategies proposed by the Human Immune Consortium (12) and are described in the online supplement (*see* Tables E1 and E2 and Figure E1 in the online supplement). Peripheral blood mononuclear cell fractions were blocked with Fc block (Miltenyi Biotec) and stained with LIVE/DEAD (Thermo Fisher Scientific) before antibody staining for 20 minutes at 4°C. Stained samples were fixed in fluorescence-activated cell sorting fixative (1% formaldehyde, phosphate-buffered saline). Samples were analyzed using a BD LSRFortessa analyzer (BD Biosciences) and analyzed using FlowJo software (version 10.0.7, BD Biosciences). Additional details of the statistical analysis and quantification of immunoglobulin concentrations and IL-21 are provided in the online supplement.

### Autoimmune Autoantibody Quantitation in Patients with IPAH and HPAH

A screen of sera from 473 patients with PAH and 946 age and sex propensity-matched healthy donor control subjects (1:2 ratio using the MatchIt package version 3.0.2) were analyzed for the presence of 19 well-characterized, autoimmune disease–associated autoantibodies using an IgG multiplex MagPlex particle-based flow cytometry assay; the ProtoPlex Autoimmune Panel (Thermo Fisher Scientific). Briefly, 2.5 μl serum was incubated with MagPlex microspheres conjugated to 19 multiplexed human autoimmune antigens, following the manufacturer’s instructions, followed by data acquisition using a Luminex 200 system (Luminex). Median fluorescence indices were corrected for background signal and nonspecific binding using an internal bovine serum albumin protein control before processing. Differences in autoantibody positivity between patients and control subjects were assessed (on the basis of the distribution of autoantibody concentrations in the control population). Subsequently, after determining the optimal clustering algorithm and number of clusters (K), autoantibody samples of patients and control subjects were clustered using partitioning around medoids with *K* =3. Only autoantibody samples and not clinical characteristics were included in the clustering to prevent bias. Differences in autoantibody positivity, clinical characteristics, and mortality between clusters were assessed using chi-square tests, ANOVAs and analysis of covariance, Kruskal-Wallis tests, and log-rank tests. Full details of the statistical analysis and clustering methods are provided in the online supplement.

Additional details of the methodology for detection of BMPR2 autoantibodies, including glutathione S-transferase fusion microarray screen, BMPR2 autoantibody ELISA, and pulmonary arterial smooth muscle cell culture, are provided in the online supplement.

## Results

### Patients with IPAH Have an Immune Phenotype Defined by a Shift in the Adaptive Immune Response Axis

Circulating leukocyte populations were studied using flow cytometry in patients with IPAH (*n* = 26; mean age, 41.3 ± 10.2 yr), selected with no known PAH-causing genetic mutations to limit confounding influences of existing pathologies and compared with age- and sex-matched healthy donors (*n* = 29). Demographic and clinical characteristics are described in Table 1. Antibody staining panels correlated well for lymphocyte gates and major T-cell populations across panels (*see* Figure E4).

**Figure 1:**
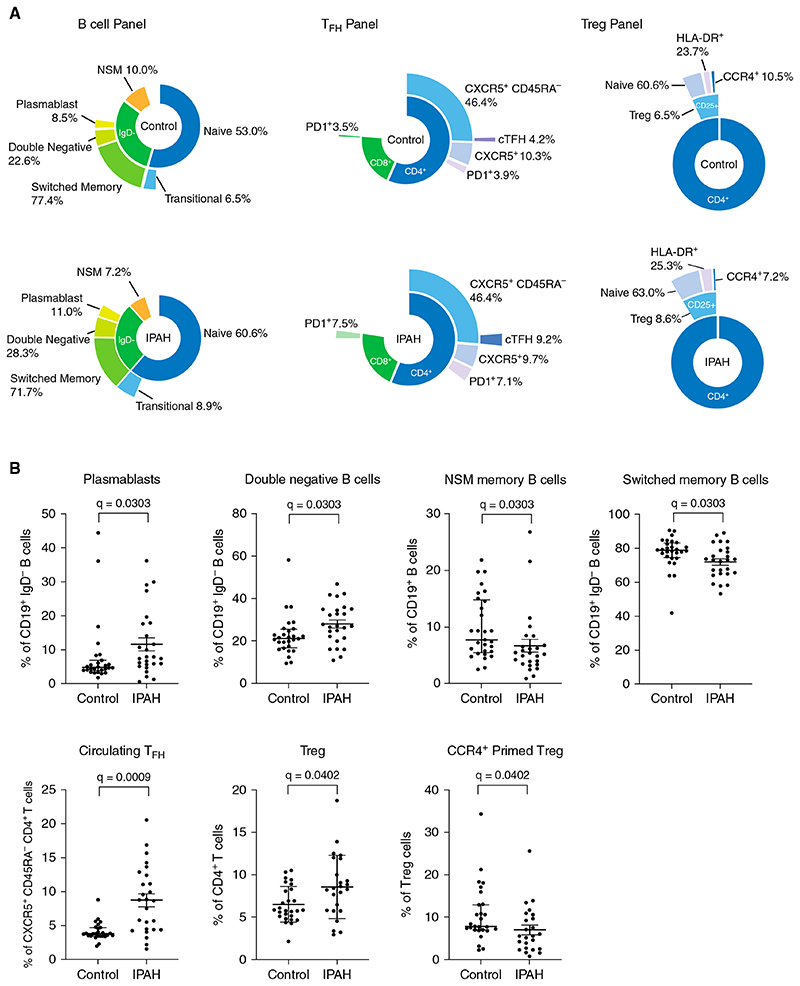
The peripheral immune profile in idiopathic pulmonary arterial hypertension (IPAH) is one of an activated immune response. Circulating leukocytes from peripheral blood mononuclear cell fractions of peripheral whole blood were analyzed in subjects with IPAH (*n* = 26) and healthy donor control subjects (*n* = 29) using flow cytometry. (*A*) Sunburst plots showing average distribution of B-cell, T follicular helper (T_FH_) cell, and regulatory T (Treg) cell populations as a percentage of parent population from their respective panels of CD19^+^ B cells, T helper/T_FH_ cells, and CD4^+^ T cells in healthy donors and subjects with IPAH. (*B*) Abundance of cell populations for plasmablasts (CD3^−^ CD19^+^ CD38^+^ IgD^−^), double-negative B cells (CD3^−^ CD19^+^ CD38^−^ CD27^−^ IgD^−^), NSMs (CD3^−^ CD19^+^ CD27^+^ IgD^+^), switched memory B cells (CD3^−^ CD19^+^ CD38^+^ CD27^+^ IgD^−^), “circulating” T_FH_ cells (CD3^+^ CD4^+^ CXCR5^+^ CD45RA^−^ PD-1^+^), Treg cells (CD3^+^ CD4^+^ CD25^+^ CD127^−^), and CCR4^+^-primed Treg cells (CD3^+^ CD4^+^ CD25^+^ CD27^−^ CCR4^+^). Plots show median with interquartile range except for Treg cells (mean with SD). To control for multiple hypothesis testing, false discovery rates were estimated using the Benjamini-Hochberg procedure on a per-panel basis, and resultant *q* values are presented. We report here as significant tests *q*< 0.05. CD = cluster of differentiation; cTFH = circulating T follicular helper; CXCR5 = C-X-C chemokine receptor type 5; NSM = nonswitched memory B cell; PD1 = programmed cell death protein 1.

IPAH was characterized by a distinct immune profile of altered B-cell frequencies, increased circulating T follicular helper (T_FH_) cells, and an altered regulatory T (T_REG_)–cell profile, an immune phenotype indicative of an activated immune response (Figure 1A). We identified significantly enriched populations of plasmablasts (CD3 [cluster of differentiation 3] CD19^+^ CD38^+^ IgD^−^) and double-negative B cells (CD3^−^ CD19^+^ CD38^−^ CD27^−^ IgD^−^) and comparative decreases in nonswitched memory B-cell (CD3^−^ CD19^+^ CD27^+^ IgD^+^) and switched memory B-cell (CD3^−^ CD19^+^ CD38^+^ CD27^+^ IgD^−^) frequencies (Figure 1B) in IPAH. A significant increase in “circulating” T_FH_ cells (CD3^+^ CD4^+^ CXCR5^+^ [C-X-C chemokine receptor type 5] CD45RA^−^ PD-1^+^ [programmed cell death protein 1]) was observed together with increased amounts of T_REG_ cells (CD3^+^ CD4^+^ CD25^+^ CD127^−^), with a comparative reduction in CCR4^+^ (C-C chemokine receptor 4)-primed TREG cells (CD3^+^ CD4^+^ CD25^+^ CD27^−^ CCR4^+^) (Figure 1B). Full results of immunophenotyping can be found in Table E3. Frequencies of T_REG_ cells correlated with increased frequency of circulating T_FH_ cells and plasmablasts, and plasmablast concentrations negatively correlated with nonswitched memory B-cell frequencies, suggesting an interplay between the activity of immune cell subpopulations (*see* Figure E5).

### Immunoglobulin IgG3 and IL-21 Are Increased in IPAH

**Figure 2:**
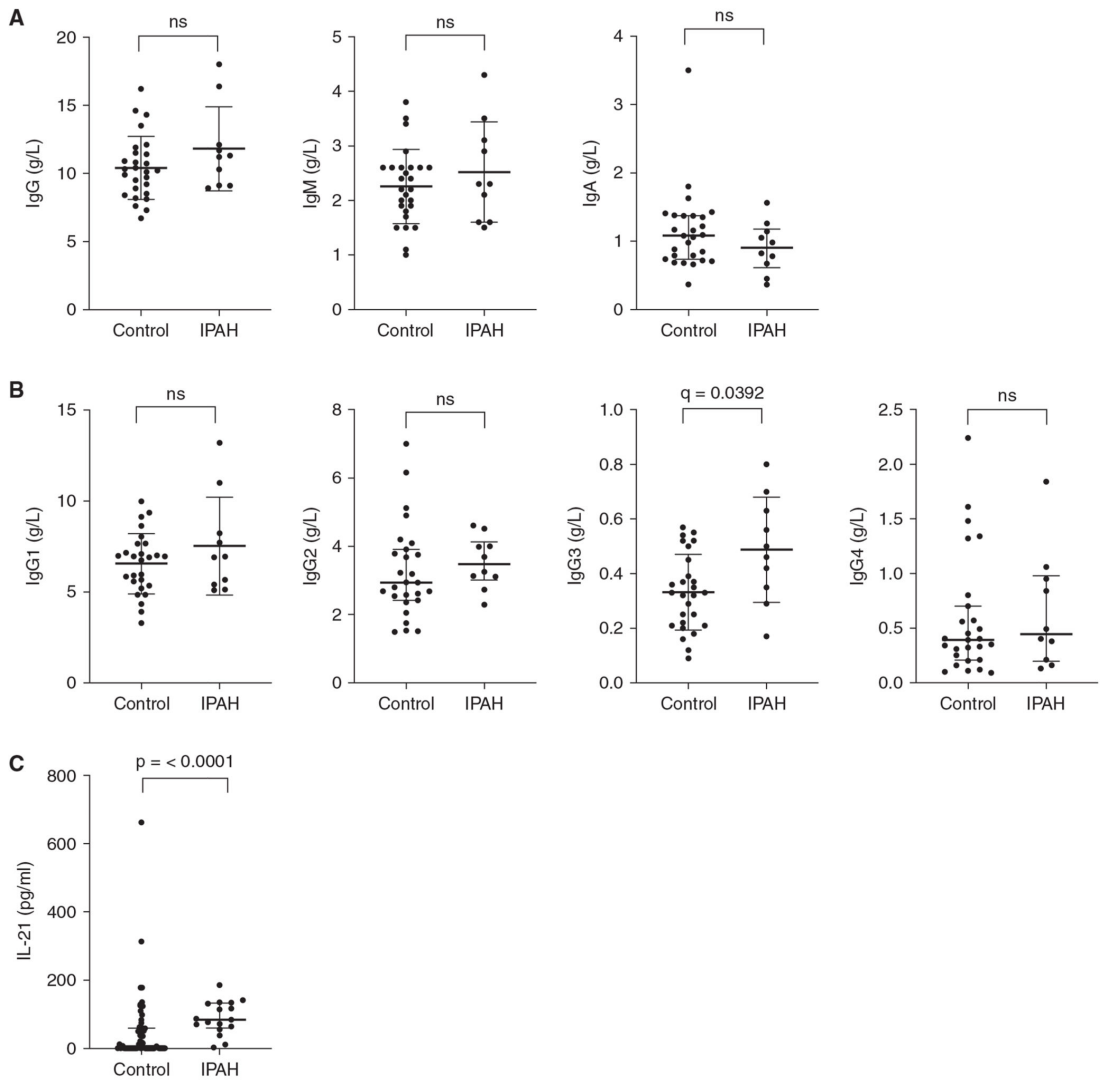
Circulating immunoglobulin and IL-21 concentrations in subjects with idiopathic pulmonary arterial hypertension (IPAH). (*A* and *B*) Nephelometry of peripheral immunoglobulin classes in subjects with IPAH (*n* =10) and healthy donor control subjects (*n* = 27) for (*A*) major immunoglobulin classes (data shown as mean with SD for IgG and IgM and as median with interquartile range for IgA) and (*B*) IgG subclasses (data shown as mean with SD for IgG1 and IgG3 and as median with interquartile range for IgG2 and IgG4). Patients with IPAH were selected according to the availability of plasma samples from the immunophenotyping cohort. To control for multiple hypothesis testing, false discovery rates were estimated using the Benjamini-Hochberg procedure for a total of four tests, and resultant *q* values are presented. We report here as significant tests *q*< 0.05. (*C*) IL-21 concentrations in subjects with IPAH (*n* = 17) and healthy donor control subjects (*n* = 60). Data are shown as median with interquartile range with Mann-Whitney test. ns = not significant.

Circulating concentrations of immunoglobulin were subsequently measured in a random subset of patients with IPAH with available plasma from the immunophenotyping analysis (*n* = 10). Although major subclasses remained unchanged compared with healthy control subjects (Figure 2A), IgG3 concentrations were increased in subjects with IPAH (*q* = 0.0392; Figure 2B). IL-21, which has not been previously measured in IPAH, plays a major role in B-cell immunoglobulin response and has been implicated in the promotion of autoimmune disease (13). IL-21 concentrations were shown to be significantly increased in IPAH (*P* < 0.0001; Figure 2C).

### Autoantibody Concentrations Are Increased in IPAH and HPAH

**Figure 3:**
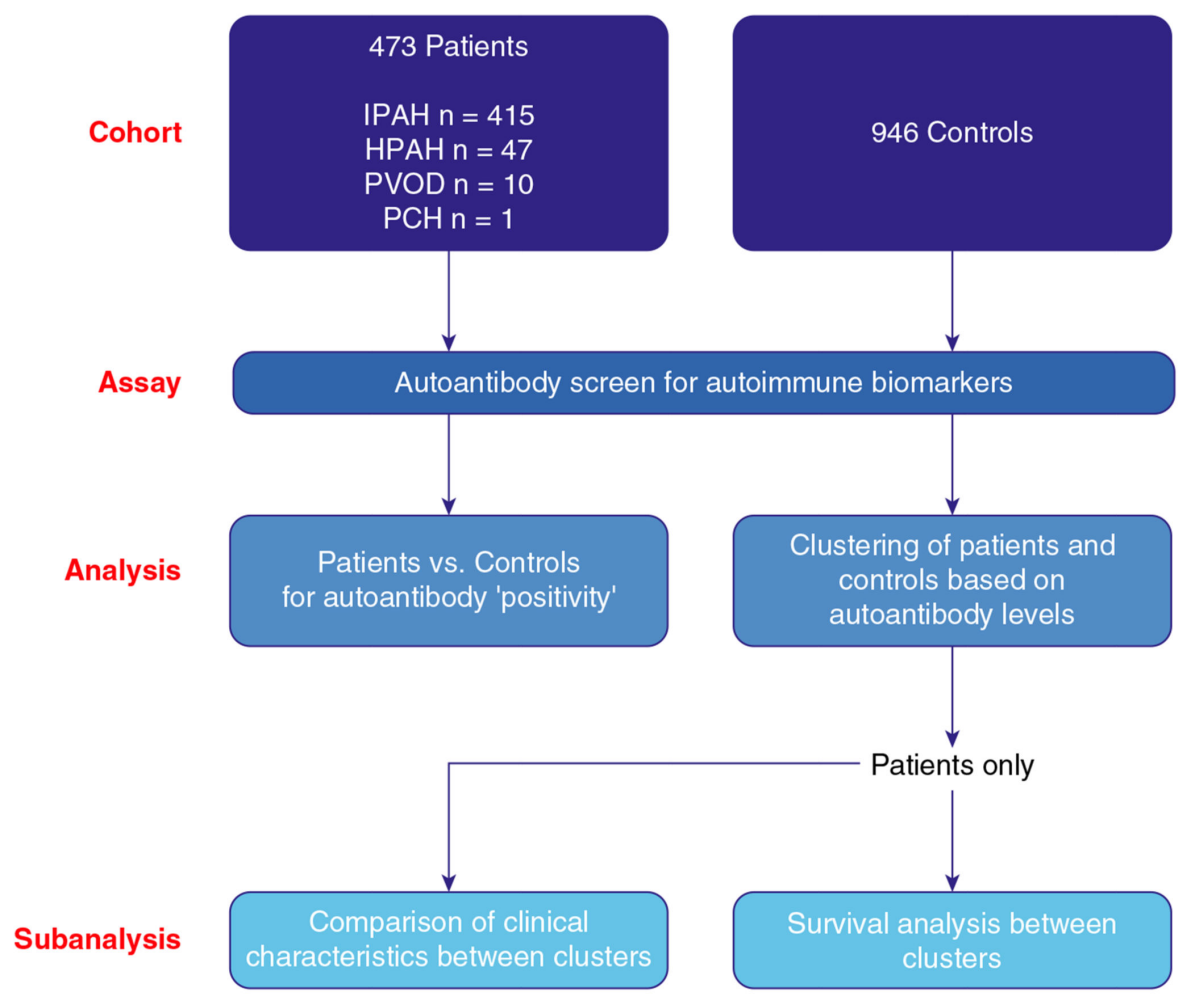
Strategy for the detection of autoantibody biomarkers in subjects with pulmonary arterial hypertension (PAH) and healthy donor control subjects and subsequent cluster analysis for the evaluation of clinical characteristics in subjects with PAH on the basis of autoantibody status. HPAH = heritable PAH; IPAH = idiopathic PAH; PCH = pulmonary capillary hemangiomatosis; PVOD = pulmonary venoocclusive disease.

Our data are indicative of an aberrant immune phenotype in IPAH that is suggestive of autoimmune pathology. To assess the prevalence of autoantibodies in PAH, we used the ProtoPlex Autoimmune Panel to screen for 19 clinically standard and widely used autoantibody biomarkers in sera from a cohort of subjects with IPAH/HPAH/pulmonary venoocclusive disease/pulmonary capillary hemangiomatosis (*n* = 473). These were compared with an age and sex propensity-matched healthy donor control cohort (*n* = 946) (*see* Tables 2 and E4 for demographic and clinical characteristics). The analysis plan is summarized in Figure 3 and was devised to *1*) compare PAH and healthy donor control autoantibody positivity; *2*) identify clusters of patients on the basis of autoantibody profile; and 3) stratify clinical outcomes and prognosis on the basis of clustering.

**Figure 4:**
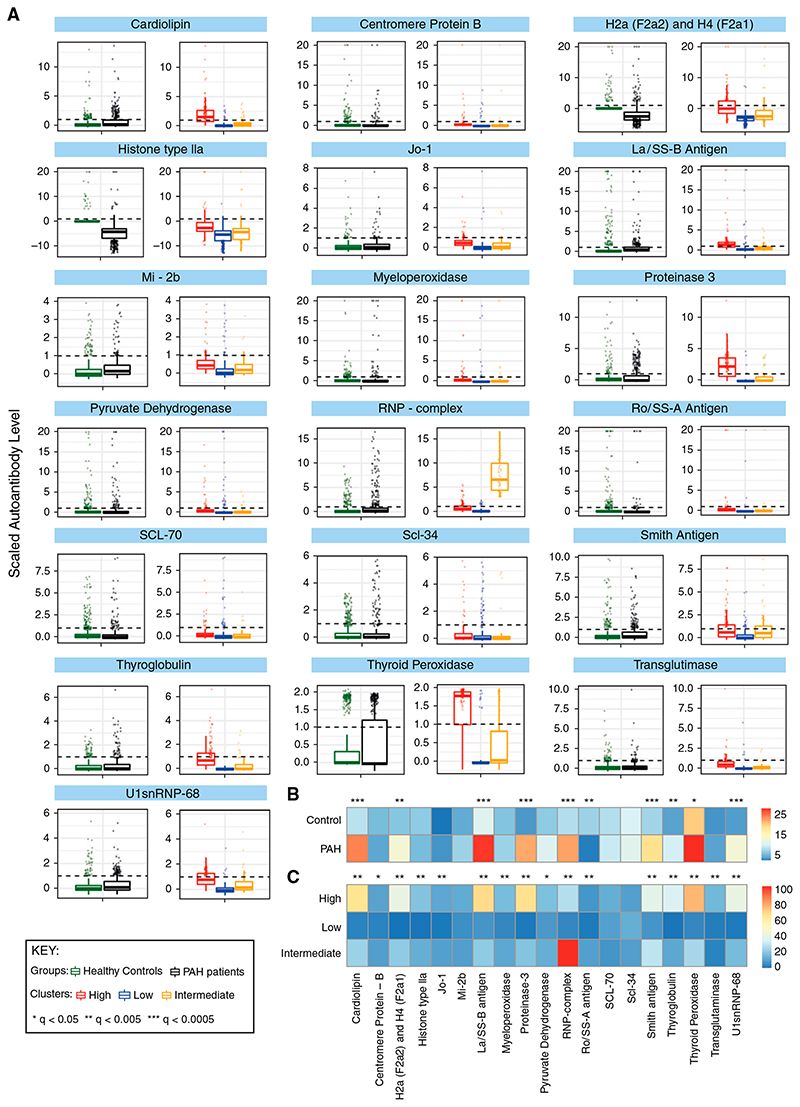
Autoantibody analysis in pulmonary arterial hypertension (PAH). Circulating autoantibodies were assayed using the ProtoPlex Autoimmune Panel in 473 patients with PAH and 946 age- and sex-matched healthy control subjects. (*A*) Autoantibody concentrations were compared in both healthy control subjects and patients with PAH (left-side graphs) and among clusters of patients with PAH (right-side graphs). Positivity was defined as 0.75Q + 2IQR of the control population (shown as a dashed line set at 1 for the normalized autoantibody concentration). Box plots show the median and IQR (25%, 75%); whiskers represent the end of the box plot ± 1.5 × IQR. The y-axis represents the scaled autoantibody concentration, which is normalized to have a median of 0 in the control population and a positivity threshold of 1. (*B*) Heat map showing autoantibody positivity prevalence as a percentage in cases with PAH and control subjects. FDR-adjusted *q* values were calculated across 19 tests and are indicated using asterisks (*q<0.05, **q<0.005, and ***q<0.0005). (*C*) Heat map showing autoantibody positivity prevalence among clusters of cases with PAH: high, low, and intermediate. FDR-adjusted *q* values were calculated across 19 tests and are indicated using asterisks and indicate which autoantibodies are driving the clustering. Note that the null hypotheses of no difference among clusters are not sensible to test, because clusters were defined on the basis of observed values. *B* and *C* summarize the significance testing results shown in detail in A. FDR = false discovery rate; IQR = interquartile range; La/SS-B = La antigen; RNP = ribonucleic protein; Ro/SS-A = Ro antigen; SCL = scleroderma antigen; U1snRNP-68 = U1 small nuclear ribonucleic protein 68.

Autoantibody positivity differed significantly between PAH and healthy donor control subjects (Figure 4A, left-hand graphs, and Table E5); 10 autoantibodies differed significantly between groups, of which 9 (cardiolipin, histones H2a [F2a2] and H4 [F2a1], La/SS-B antigen, proteinase-3, RNP [ribonucleic protein] complex, Smith antigen, thyroglobulin, thyroid peroxidase, and U1-snRNP [U1 small nuclear RNP] 68) were more commonly positive in PAH (Figure 4B).

### Cluster Analysis Reveals Distinct Subgroups According to Autoantibody Profile

Three distinct clusters according to autoantibody concentrations in the combined healthy donor control and PAH cohorts were identified using partitioning around medoids clustering, stratifying into a “high-autoantibody” cluster, a “low-autoantibody” cluster, and a small “intermediate” cluster exhibiting high concentrations of RNP-complex autoantibodies. Stratification of patients with PAH alone resulted in a similar clustering pattern according to autoantibody positivity, with 27.5% in the high cluster (*n* = 130), 61.3% in the low cluster (*n* = 290), and 11.2% in the intermediate cluster (*n* = 53) (Figure 4A, right-hand graphs; Figure 4C, heat map; and Table E6). Subsequent analysis of clusters within patients with PAH alone revealed that distributions of age at diagnosis and sex were comparable among the clusters (see Table E7).

### PAH Autoantibody Clusters Have Distinct Clinical and Mortality Outcomes

**Figure 5:**
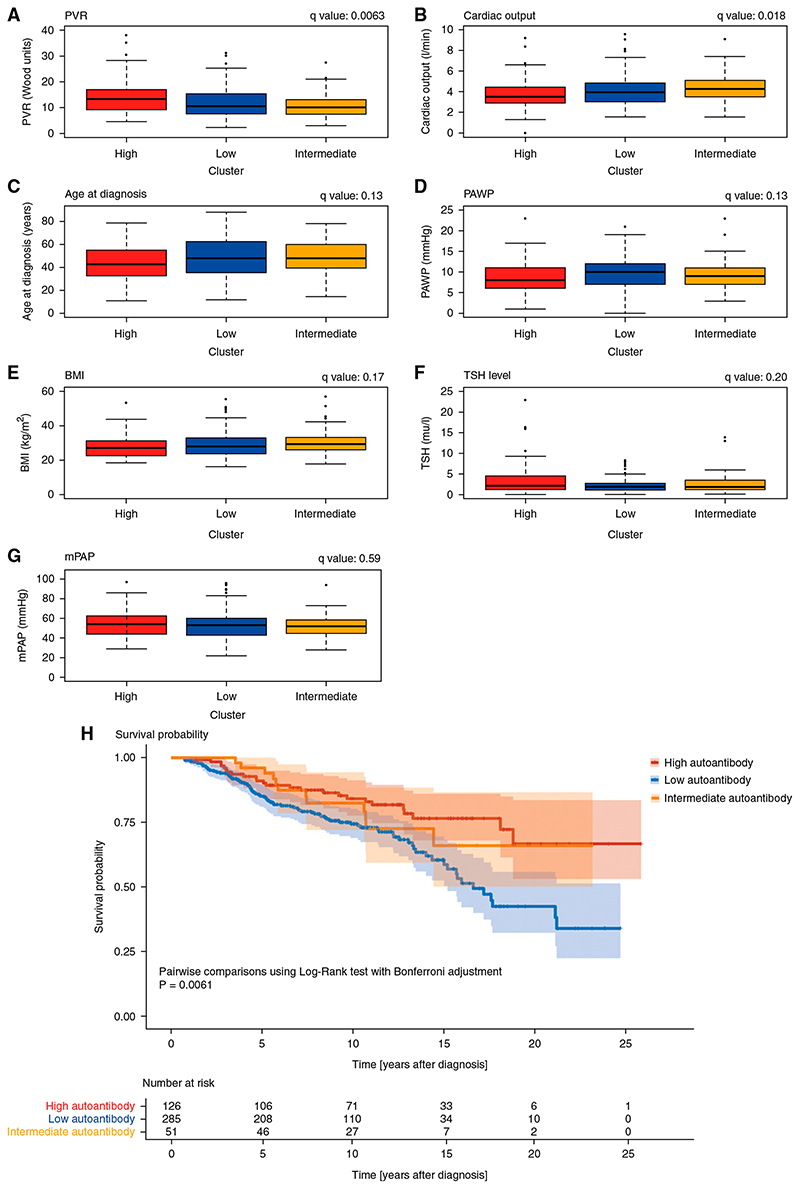
Clinical comparison of clusters in pulmonary arterial hypertension (PAH). (*A–G*) Box plot comparison of stratification of clinical outcomes in the PAH cohort as determined using autoantibody cluster analysis for (*A*) PVR, (*B*) cardiac output, (*C*) age at diagnosis, (*D*) PAWP, (*E*) BMI, (*F*) thyroid-stimulating hormone (TSH) concentration, and (*G*) mPAP. Box plots represent the median with IQR (25th-75th percentile); whiskers represent the end of the box plot ± 1.5 × IQR. Statistical analysis was performed on all available values using two-tailed ANOVA with false discovery rate-adjusted *q* values calculated across 178 tests. TSH concentrations were log transformed before the ANOVA. (*H*) Kaplan-Meier survival analysis for patients up to 20 years after diagnosis (*n* = 462). Statistical analysis was performed using pairwise log-rank tests with a global log-rank test on 2 degrees of freedom. BMI = body mass index; IQR = interquartile range; mPAP = mean pulmonary arterial pressure; PAWP = pulmonary arterial wedge pressure; PVR = pulmonary vascular resistance.

Comparison of clinical characteristics among patients with PAH defined by their autoantibody clusters identified significant differences in hemodynamic parameters (Figure 5 and Table E7). PVR and cardiac output varied significantly among clusters (*q* = 0.0063 and *q* = 0.018, respectively) and were worse in the high-autoantibody cluster (PVR, 14.0 ± 6.5 vs. 11.7 ± 5.4 and 10.8 ± 5.1 Wood units; cardiac output, 3.7 ± 1.3 vs. 4.1 ± 1.3 and 4.4 ± 1.5 L/min; high-autoantibody cluster vs. low-autoantibody and intermediate-autoantibody clusters, respectively). No significant differences in pulmonary arterial wedge pressure or mean pulmonary arterial pressure were observed among clusters (Figures 5D and 5G), nor were any immunemediated comorbidities or clinical indications of autoimmunity. The high- and intermediate-autoantibody clusters were found to have greater prevalence of comorbid hypothyroidism (25.4%, 7.9%, and 20.8% for the high, low, and intermediate clusters, respectively), but no differences were identified for thyroid-stimulating hormone concentrations (*q* = 0.20; Figure 5F). Age and body mass index did not affect cluster stratification (Figures 5C and 5E). No significant differences in treatment were observed among clusters (*q* = 0.12). REVEAL (Registry to Evaluate Early and Long-Term Pulmonary Arterial Hypertension Disease Management) risk score did not differ statistically among clusters (*q* = 0.25), but significant variation in WHO functional class was observed among clusters (*q* = 0.042); the high-autoantibody cluster had a higher proportion of subjects in WHO functional class IV (20.0% vs. 11.3% [low cluster] and 1.9% [intermediate cluster]) and comparatively fewer subjects in class III (51.5% [high cluster] vs. 67.6% [low cluster] and 71.7% [intermediate cluster]). Kaplan-Meier survival curves using census data from the patients sampled in the UK National Cohort Study of Idiopathic and Hereditary PAH differed among clusters (*P* = 0.0061, log-rank test; Figure 5H). This demonstrated improved survival at 20 years after diagnosis among groups and indicated highest survival in the high-autoantibody cluster, and after correction for treatment, sex, and age at diagnosis in a Cox proportional-hazard model (Figure E6), subjects in the low-autoantibody cluster remained at significantly increased risk for mortality (odds ratio, 1.9; 95% confidence interval, 1.17−3.0).

### Putative Autoantibodies to BMPR2 Are Detected in IPAH Sera

**Figure 6:**
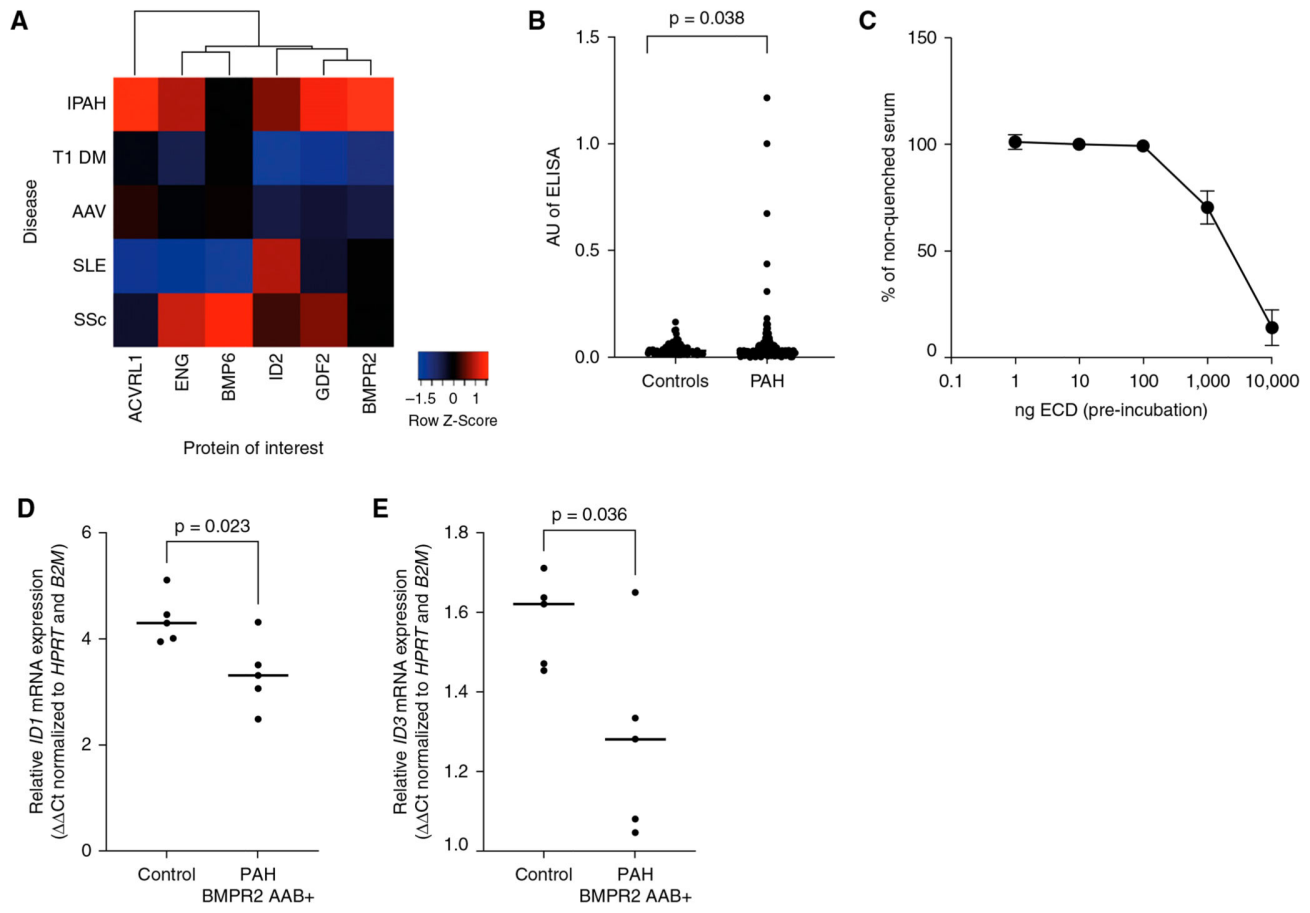
Putative antibodies to BMPR2 (bone morphogenetic protein receptor 2) are present in sera of patients with pulmonary arterial hypertension (PAH). (*A*) Heat map showing reactivity after a glutathione S-transferase fusion human proteomic screen to identify sera reactivity to proteins in the BMPR pathway. Sera were obtained from patients with idiopathic PAH (IPAH) (*n* = 5) and with comparator autoimmune diseases (T1 DM, *n* = 3; AAV, *n* = 3; SLE, *n* = 2; SSc, *n* = 3). Heat map shows mean reactivity (arbitrary units). (*B*) Quantitative analysis of IgG reactivity against a recombinant peptide of the BMPR2 extracellular domain (ECD) in sera from patients with IPAH/heritable PAH (n =350) and healthy donors (*n* = 55). The Mann-Whitney test was performed between control subjects and PAH groups. (*C*) Quenching of identified seropositive samples (*n* = 5) with free ECD before incubation on ELISA effectively quenches binding at >1,000 ng. Data are shown as mean with SD and expressed as a percentage of nonquenched serum. (*D* and *E*) Effect of serum preincubation from control subjects (*n* = 5) or patients with PAH with seropositivity to BMPR2 as shown by ELISA (n =5) on pulmonary arterial smooth muscle cells for 1 hour, followed by stimulation with 10 ng/ml BMP4 for 1 hour. Relative quantification of downstream *ID1* (*D*) and *ID3* (*E*) by quantitative PCR and normalized to *HPRT* and *B2M*. AAB = autoantibody; AAV = antineutrophil cytoplasmic antibody–associated vasculitis; ACVRL1 = activin A receptor like type 1; AU = arbitrary units; *B2M* = β-2-microglobulin; BMP = bone morphogenetic protein; ENG = endoglin; GDF2 = growth differentiation factor 2; *HPRT* = hypoxanthine phosphoribosyltransferase 1; ID = inhibitor of DNA binding protein; SLE = systemic lupus erythematosus; SSc = systemic sclerosis; T1 DM = type 1 diabetes mellitus.

Using a candidate-based glutathione S-transferase fusion human proteomic screen, we tested the hypothesis that BMPR2 and members of its canonical signaling pathway would represent “high value” targets in an autoantibody immune response in PAH (10). Putative serum autoantibodies against BMPR2 were detected in patients with IPAH (*n* = 5) but not in patients with other autoimmune etiologies (SLE, antineutrophil cytoplasmic antibody−associated vasculitis, type 1 diabetes, and SSc; *n* = 11; Figure 6A). We proceeded to screen 350 patients with IPAH and HPAH from the UK National Cohort Study of Idiopathic and Heritable PAH and 55 healthy donor control subjects in a novel ELISA to detect IgG reactivity against a peptide of the extracellular domain of human BMPR2. Bound immunoglobulins were significantly increased in patients with PAH (P = 0.038), but they were present in only a small subset of the subjects with PAH who exhibited high BMPR2 reactivity (Figure 6B). Although samples tested here overlapped with autoantibody biomarker analysis, we identified no significant enrichment into the current autoantibody clusters, though the sample size is small, and this finding should not be overinterpreted. Preincubation of positive sera with BMPR2 extracellular domain before incubation in the ELISA demonstrated a dose-responsive quenching of signal but with high concentrations for maximal effect (Figure 6C). Functional attenuation of BMP4 (bone morphogenetic protein 4) signaling in pulmonary arterial smooth muscle cells was observed with serum pretreatment from BMPR2-seropositive patients, compared with serum from healthy control subjects. Seropositive samples were shown to significantly reduce the upregulation of *ID1* (inhibitor of DNA binding protein 1) and *ID3* (inhibitor of DNA binding protein 3) after BMP4 stimulation indicating a reduction in BMPR2–ALK3 (activin receptor–like kinase 3) receptor pathway signaling (Figures 6D and 6E).

## Discussion

We present data from the largest study to date to look in depth at autoimmunity in IPAH. There is clear and consistent evidence across multiple methodologies to suggest that autoimmunity is a clinically important associative feature of IPAH.

The IPAH peripheral blood immune profile was characterized by increased concentrations of circulating T_FH_ and T_REG_ cells and altered B-cell frequencies. Our observation of increased T_REG_ cells reaffirms similar findings in PAH (14), and our observed increases in circulating TFH-like cells are a hallmark of autoimmune disease (15, 16). Increased plasmablasts recapitulate similar results (17), but the findings of increased double-negative and decreased memory B cells are novel to IPAH and likely due in part to the underreporting of B-cell populations. Our observed increase in the frequency of antibody-producing plasmablasts and double-negative B cells, and corresponding reduction in memory B cells, may indicate a shift in the humoral immune axis in our patients in favor of an activated autoimmune state, as observed in SLE (18), SSc (19), and Hashimoto’s thyroiditis (20). Increased circulating TFH cells are known to play a role in promoting the differentiation of naive B cells into antibody-producing plasma cells, inducing secretion of immunoglobulin, and negatively correlate with memory B cells in various autoimmune diseases (15, 16), and aberrant T_REG_ cells demonstrate a loss of control of immune tolerance (21). The finding of increased IL-21 in sera provides further weight to immune disruption, as in addition to the well-documented increase in IL-6 in PAH, which has been linked to prognosis (22,23), IL-21 has pleiotropic effects on both T-cell and humoral responses. There is accumulating evidence that high concentrations result in autoimmune disturbances, giving rise to aberrant concentrations of T_FH_, T_REG_, memory B cells, plasmablasts, plasma cells, and autoantibody production (13), and may be associated with our observation of increased IgG3 (24, 25).

Multiple reports have identified circulating autoantibodies in PAH with some specificity to the vascular environment (17, 26, 27), but this is the first screen of its kind to measure clinically validated and accepted autoantibodies associated with autoimmune disease. Nine autoantibodies were highly significantly increased compared with healthy control subjects. It is notable that ~40% of our population can be considered to have “positive” autoantibodies using our conservative thresholding across the clusters (with a very small fraction in addition with BMPR2 antibodies). IPAH clustered into three populations with high and low autoantibody prevalence and a smaller intermediate subset defined by high anti–RNP-complex autoantibodies. The latter cohort is strikingly different in autoantibody profile, with 100% of all classified subjects having isolated positivity to the RNP-complex antibody only, and in the absence of the clinical features of related autoimmune diseases. Secondary analysis of patient clusters identified distinctions in hemodynamic characteristics that were defined by autoantibody prevalence, as cardiac output, PVR, and WHO classification were worse in the high-autoantibody cluster. Paradoxically, this cluster had improved long-term survival, which was unaffected by sex, age, and medication. This finding is intriguing but consistent with previous genetic data in an international genome-wide association study in which an association with specific HLA–DPB1 (DP β 1 chain) alleles is a risk factor for developing PAH but also associated with improved survival outcomes (8) and whole-blood transcriptomics that identify HLA gene expression as drivers of survival-associated endophenotypes (9). This uncoupling of hemodynamics and longer-term outcomes is important and counter to our current understanding of disease progression. The critical question remains whether autoimmunity/inflammation is a cause of PAH, a response to PAH (for example, in response to pulmonary vascular cell damage), or a confounding variable that modulates response. Irrespective of this question, the demonstration of an HLA locus and now antibody signatures that both associate with better outcomes needs careful consideration. Further work is required to study the effects of patients longitudinally to assess if inflammation/autoimmunity is consistent in a subset of the disease or if it follows a profile of relapsing/remitting.

Associations of immune clusters with treatment may need to be reconsidered for the purpose of future trials. Recent work by our group failed to demonstrate a treatment effect in a stable group of unstratified patients with PAH using the anti-IL-6 drug tocilizumab on the basis of hemodynamic outcomes (28). In a *post hoc* study, Zamanian and colleagues (29) used machine learning to associate serum concentrations of rheumatoid factor, IL-12, and IL-17 with the greatest improvement in patients with SSc-associated PAH treated with the anti-CD20 drug rituximab in a trial designed to study the effect of B-cell depletion on disease pathology and prognosis. Pertinently, the authors acknowledged that the use of molecular phenotyping can identify potential biomarkers to predict the responsiveness of treatments to allow enrichment for optimal clinical trial patient selection. These studies directly challenge how targeting inflammation may need refinement from the perspective of whom we target and at what point in the disease course.

In our final line of inquiry, we examined whether serum from patients contained autoantibodies to the key genetic pathway pivotal in PAH biology: the BMPR2 pathway. Pathogenic anti-endothelial antibodies have been previously described in PAH, but none with affinity to proteins relevant to genetic causes of disease (17, 27). We report on a putative autoantibody to the pathological “high value” target, BMPR2, and evidence of an *in vitro* functional response on pulmonary vascular cells with reduced downstream canonical signaling with BMP ligand stimulation. The possibility of functional autoantibodies directed at key vascular cell pathways is important both pathobiologically and in considering responses to therapies, for example, targeting the BMPR2 pathway, sotatercept being the most advanced and currently in phase III studies (30). It is not clear yet how these less-common autoantibodies relate to the clusters we have observed.

## Conclusions

We present the most comprehensive study of autoimmunity in IPAH and demonstrate strong evidence for an association with the disease. Autoimmunity showed association with both hemodynamic parameters and clinical outcomes, and the demonstration of autoantibodies to BMPR2 opens up the possibility that autoantibodies are targeting key pathways leading to vascular dysfunction. Studies are required to understand the longitudinal variability, and future clinical trials may need to consider autoimmunity and inflammation in stratification techniques.

## Supplementary Material

Supplementary File 1

## Figures and Tables

**Table 1:** Characteristics of Immune Phenotyped Patients with Idiopathic Pulmonary Arterial Hypertension and Healthy Donor Control Subjects

	Healthy	IPAH
Number of subjects	29	26
Age at sampling, yr	41.5 ± 12.5	41.3 ± 10.2
Male:female	5:24	5:21
BMI, kg/m^2^	24.6 ± 3.6	27.9 ± 6.1
Number of smokers	1	1
Autoimmune comorbidities	0	5
Graves’ disease	0	4
Hypothyroidism	0	1
Age at diagnosis, yr	N/A	34.7 ± 12.0
WHO classification	N/A	
I	N/A	5
II	N/A	9
III	N/A	12
6MWD, m	N/A	464.8 ± 113.2
Hemodynamics		
mPAP, mm Hg	N/A	50.3 ± 11.8
mRAP, mm Hg	N/A	8.2 ± 3.3
PVR, Wood units	N/A	702.6 ± 334.8
BNP, pg/ml	N/A	387.5 ± 625.5
PAH-targeted treatment		
PDE5 inhibitor	N/A	16
ERA	N/A	9
Prostanoid	N/A	14
CCB	N/A	5
Riociguat	N/A	1
Diuretic	N/A	10

*Definition of abbreviations:* 6MWD = 6-minute-walk distance; BMI = body mass index; BNP = brain natriuretic peptide; CCB = calcium channel blocker; ERA = endothelin receptor antagonist; IPAH = idiopathic pulmonary arterial hypertension; mPAP = mean pulmonary arterial pressure; mRAP = mean right arterial pressure; N/A= not applicable; PAH = pulmonary arterial hypertension; PDE5 = phosphodiesterase-5; PVR = pulmonary vascular resistance; WHO = World Health Organization. Study groups were matched according to age and gender. Data are presented as counts, mean ± SD, or raw counts.

**Table 2:** Demographic Characteristics of Patients with Pulmonary Arterial Hypertension and Matched Healthy Donor Control Subjects Analyzed for Autoimmunity Autoantibody Biomarkers

	Healthy	PAH
Number of subjects	946	473
Age at sampling, yr	53.01 ± 13.4	53.05 ± 15.6
Male:female	282:664	141:332
BMI,^*^ kg/m^2^	—	28.84 ± 7.0

*Definition of abbreviations:* BMI = body mass index; PAH = pulmonary arterial hypertension. Data are presented as counts, mean ± SD, or raw counts.

* Data for BMI were missing or not available in 24 subjects.

## At a Glance Commentary

### Scientific Knowledge on the Subject

Although pulmonary arterial hypertension (PAH) is known to have a multitude of causes, the cause of idiopathic PAH (IPAH) and its association with autoimmunity have been topics of debate.

### What This Study Adds to the Field

To our knowledge this is the largest ever study of the significance of autoimmunity in IPAH and heritable PAH. We show that using multiple methodological approaches, IPAH displays an autoimmune signature. The association of autoimmunity with IPAH has important therapeutic implications.
