# Supplementary material for: Autoimmunity Is a Significant Feature of Idiopathic Pulmonary Arterial Hypertension
Source: Am J Respir Crit Care Med. Author manuscript; Available in PMC 2022 Dec 12. (PMC7613913; doi:10.1164/rccm.202108-1919OC)
Supplement: Supplementary File 1 [file EMS157109-supplement-Supplementary_File_1.pdf]

Online Data Supplement

Autoimmunity is a Significant Feature of Idiopathic Pulmonary Arterial Hypertension

Rowena J. Jones; Eckart M.D.D. De Bie; Emily Groves; Kasia I .Zalewska, Emilia M. Swietlik; Carmen M. Treacy; Jennifer M. Martin; Gary Polwarth; Wei Li; Jingxu Guo; Helen E. Baxendale; Stephen Coleman; Natalia Savinykh; J. Gerry Coghlan; Paul A. Corris; Luke S. Howard; Martin K. Johnson; Colin Church; David G. Kiely; Allan Lawrie; James L. Lordan; Robert V. Mackenzie Ross; Joanna Pepke Zaba; Martin R. Wilkins; S. John Wort; Edoardo Fiorillo; Valeria Orrù; Francesco Cucca; Christopher J. Rhodes; Stefan Gräf; Nicholas W. Morrell; Eoin F. McKinney; Chris Wallace; Mark Toshner

The UK National PAH Cohort Study Consortium

Supplementary Methods

Statistical analysis of flow cytometric standardised immunophenotyping.

Statistical comparisons were performed using GraphPad Prism version 8. Analysis of the detailed immune profiles of patients was performed on frequencies of cell populations represented as a percentage of the parent population. Patient and healthy donor groups were compared using unpaired t-test or Mann-Whitney test based on data normality as determined by D'Agostino& Pearson test for normality. To control for multiple hypothesis testing, false discovery rates were estimated using Benjamini and Hochberg on a per panel basis, and resultant q values are presented. That is, FDR was run separately for the sets of tests displayed within each panel of Figure 1, the whole of Figure 2, Figure 4B, Figure 4C and Figure 5. We report here as significant tests $q<0.05$. Correlation analysis was performed using two-tailed Pearson correlation co-efficient or Spearman correlation dependent upon normality of the data. Sunburst plots were generated in Microsoft Excel.

Quantification of immunoglobulin levels and interleukin-21

Peripheral blood serum samples were prepared and stored at -80°C prior to analysis. Standard nephelometry was used to determine absolute amounts of immunoglobulin subclasses present in sera. Levels of serum IL-21 were quantified using the Human IL-21 Ready-SET- Go ELISA (eBioscience).

Descriptive statistics of the PAH patients in the autoantibody analysis.

Differences in demographics, haemodynamic parameters and clinical indications for autoimmunity were compared between different PAH aetiologies using ANOVAs and Chi-square tests. A similar comparison was made between incident and prevalent cases, using independent samples T-tests and Chi-square tests. To control for multiple hypothesis testing,

false discovery rates were estimated using Benjamini and Hochberg within each set of 22 tests, and resultant q values are presented. We report here as significant tests $q<0.05$.

Statistical analysis of autoantibody quantitation assay.

Statistical comparisons were performed using R/R studio v3.6.3 using the stats (v3.6.3) and Publish (v2019.12.04) packages. The base (v3.6.3), MatchIt (v3.0.2), stringdist (v0.9.6.3), data.table (v1.13.0) and tidyverse (v1.3.0) packages were used for data transformations. We report here as significant tests $q<0.05$. Log_e transformed autoantibody values for 473 patients and 946 healthy donor controls were assessed for autoantibody positivity. A subject was classified as positive for an autoantibody if levels exceeded 0.75Q + 2IQR of the control population. Differences in positivity prevalence between cases and controls for each autoantibody were assessed with a Chi-square test and FDR q-values were calculated across 19 tests. Clustering was performed using PAM (partition around medoids) because we considered using medoids more robust to the non-normal distribution of some autoantibodies. Euclidean distances were used, as data was on the same scale and only one type of variable was used. We used silhouette and elbow plots to identify the optimal number of clusters (K) and determined that K=3 was optimal for this dataset (See Figure E2). Differences in autoantibody positivity proportions between clusters was assessed with a Chi-square test and FDR q-values were calculated across 19 tests. Differences between clusters of patients alone based on autoantibody levels and clinical data at diagnosis were assessed using Kruskal-Wallis, AN(C)OVA and Chi-square tests and FDR q-values were calculated across 178 tests. Comparison of clinical outcomes with autoantibody clusters was assessed using ANOVA on numeric clinical data (exception; TSH levels were log_e transformed). Where numeric clinical data differed significantly, an ANCOVA correcting for BMI, age at diagnosis, sex and aetiology was performed with Bonferroni correction for three tests. Survival was assessed using

log-rank tests based on right-censored left-truncated Kaplan-Meier curves with the survival (v3.2-3) and survminer (v0.4.8) packages for 462 patients (only patients with a time to event less than or equal to 20 years were included to reduce immortal time bias). The same packages were used to calculate survival differences (odds ratios), corrected for age at diagnosis, sex and treatment, using a Cox-proportional hazard model. Where FDR adjustment (according to Benjamini & Hochberg) was not possible i.e. for comparisons with ten or less tests, Bonferroni correction was applied. Data was visualised using the ggplot2 (v3.3.0), Pheatmap (v1.0.12) ggbeeswarm (v0.6.0), cowplot (v1.1.0), ggrastr (v0.2.1), Factoextra (v1.0.7), survival (v3.2-3), and survminer (v0.4.7) packages. The complete code used for the analyses of the PAH cohort (n=473) and healthy controls (n=946) in relation to the 19 autoantibodies can be found on: https://github.com/EckartDeBie/Autoimmunity_in_PAH.

Autoantibody microarray discovery

Autoantibody screening was undertaken using a custom protein microarray platform (HuProt™ version 2.0) platform in collaboration with Cambridge Protein Arrays Ltd. (Cambridge, UK). Protein microarrays consisted of a glass microscope slide with a thin SuperEpoxy coating, printed with triplicate spots of recombinant yeast-expressed whole proteins fused with a GST (glutathione-S-transferase) tag. The array included 19,500 targets with selected proteins in the BMPR pathway analysed for this study. Slides were blocked in PBS with 2% BSA /0.1% Tween-20 overnight at 4°C, washed, and then incubated with serum diluted 1:1000 at room temperature for two hours before washing and incubation at room temperature for two hours with fluorophore-conjugated goat anti-human IgM-μ chain-Alexa488 and goat anti-human IgG-Fc-DyLight550 secondary antibodies (Invitrogen). After further washing, slides were scanned using a Tecan LS400 scanner with images extracted using GenePix Pro v4 software. Sera from five patients with IPAH were included in addition to positive control autoantibody

driven disease samples in type one diabetes mellitus (n=3), ANCA associated vasculitis (n=3), systemic lupus erythematosus (n=2) and scleroderma (n=3).

BMPR2 Autoantibody ELISA

Putative autoantibodies to the extracellular domain (ECD) of BMPR2 were detected using a novel ELISA. BMPR2 ECD was generated as previously described (1). Serum samples were obtained from 350 IPAH/ HPAH patients and 55 healthy donor controls and stored at -80°C prior to analysis. Diluted sera were incubated overnight at 4°C on microplates that had been pre-coated with 100ng of BMPR2 ECD and subsequently exposed to a blocking reagent of PBS with 0.1% Tween-20. Serum dilution of 1:250 in PBS with 0.05% Tween-20 was initially determined (See online supplementary Figure E3A). Immunoglobulin bound to the ECD was detected using Horseradish peroxidase-conjugated anti human IgG monoclonal antibody (Invitrogen) and quantified by addition of Tetramethylbenzidine which was terminated with addition of 1M H_2PO_4 and measured at 450nm (550nm subtraction, BioRad). The results of the tested samples were normalised against a positive reference serum. All samples were tested in duplicate and coefficient of variation of inter-assay variability was tested (See online supplementary Figure E3B). To check for false positivity, IgG reactive sera were incubated in the absence of coated ECD and if still shown to be reactive were removed from the analysis (See online supplementary Figure E3C). To test the ability of free ECD to quench sera immunoglobulins from binding bound peptide, a range of 1 to 10,000 ng ECD was preincubated overnight at 4°C with sera that had demonstrated reactivity to BMPR2 ECD and then subjected to the ELISA assay.

Pulmonary arterial smooth muscle cell treatment with serum

Pulmonary arterial smooth muscle cells from healthy donors were cultured in DMEM (Thermo Fisher) with 10% FBS (Thermo Fisher) and antibiotics / antimitotics (Thermo Fisher) in 12

well plates until 80% confluence. Cells were serum starved overnight in DMEM plus 0.1% FBS. Cells were subsequently pre-incubated for one hour in 0.1% FBS DMEM with a 1 in 100 dilution of serum from either PAH patients showing BMPR2 sero-positivity in the BMPR2 ELISA (n=5), or age and sex matched healthy controls (n=5). The media was then spiked with 10ng/ml BMP4 (RnD Systems) for a further one hour. Cells were then lysed in RLT buffer (Qiagen) containing 1% beta mercaptoethanol (Sigma Aldrich). RNA was extracted using the Qiagen RNease mini kit with on-column DNase digestion. RNA was then quantified using a Nanodrop Lite (Thermo Fisher) and cDNA generated using the High-Capacity cDNA reverse transcription kit (Thermo Fisher) according to manufacturer's instructions. qPCR was then performed using primers for *ID1* (Qiagen Quantitect QT00230650), *ID3* (Qiagen Quantitect QT01673336), *B2M* (F: CTCGCGCTACTCTCTCTTTCT, R: CATTCTCTGCTGGATGACGTG) and *HPRT* (F: GCTATAAATTCTTTGCTGACCTGCTG, R: AATTACTTTTATGTCCCCTGTTGACTG G). Sybr Green PCR master mix (Thermo) and Rox (Thermo) were used in the reaction, to a final volume of 10ul. Thermal cycling conditions were according to the Sybr Green PCR master mix manufacturer's instructions. Gene expression was determined by the ddCT method and normalised to the average of *B2M* and *HPRT* housekeeping genes. All gene expression is shown as relative expression with BMP4 stimulation compared to unstimulated for that serum treatment.

Supplement References

E1. Jiang H, Salmon RM, Upton PD, Wei Z, Lawera A, Davenport AP, Morrell NW, Li W. The prodomain-bound form of bone morphogenetic protein 10 is biologically active on endothelial cells. *Journal of Biological Chemistry* 2016;291:2954–2966.

Supplementary Methods Tables and Figures

Table E1. Antibody cell surface markers used in immunophenotyping flow cytometry analysis. Panels are as follows; 1: Myeloid; 2: B cells; 3: Broad T cells; 4: T helper / follicular helper cells; 5: Regulatory T cells.

Antigen	Fluorochrome	Clone	Isotype	Supplier	Dilution	Panel
CD3	eVolve 605	OKT3	Mouse [IgG2a, κ]	eBioscience	20	1, 2, 3, 4, 5
CD11c	PE/Vio770	MJ4-27G12	Mouse [IgG2b, κ]	Miltenyi	11	1
CD14	eVolve 605	61D3	Mouse [IgG1, κ]	eBioscience	20	1
CD16	APC	CB16	Mouse [IgG1, κ]	eBioscience	20	1
CD19	APC/eFluor780	HIB19	Mouse [IgG1, κ]	eBioscience	20	1
CD20	APC/eFluor780	2H7	Mouse [IgG2b, κ]	eBioscience	20	1, 2
CD56	FITC	MEM188	Mouse [IgG2a, κ]	eBioscience	20	1
CD123	PerCP/Cy5.5	6H6	Mouse [IgG1, κ]	eBioscience	20	1
HLA-DR	eFluor450	L243	Mouse [IgG2a, κ]	eBioscience	20	1, 5
CD19	Brilliant Violet 450	HIB19	Mouse [IgG1, κ]	BD Biosciences	20	2
CD24	PerCP/Cy5.5	ML5	Mouse [IgG2a, κ]	BD Biosciences	20	2
CD27	PE/Cy7	O323	Mouse [IgG1, κ]	eBioscience	20	2
CD38	APC	HIT2	Mouse [IgG1, κ]	BD Biosciences	5	2
IgD	FITC	IA6-2	Mouse [IgG2a, κ]	BD Biosciences	5	2
CCR7	PE	150503	Mouse [IgG2a]	BD Biosciences	5	3
CCR6	PE/Cy7	G034E3	Mouse [IgG2b, κ]	Biolegend	5	3, 4
CD4	APC/eFluor 780	RPA-T4	Mouse [IgG1, κ]	eBioscience	20	3, 4, 5
CD8	eVolve 655	RPA-T8	Mouse [IgG1, κ]	eBioscience	20	3, 4
CD45RA	PerCP/Cy5.5	HI100	Mouse [IgG2b, κ]	eBioscience	20	3, 4, 5
CXCR3	FITC	49801	Mouse [IgG1, κ]	R&D Systems	20	4
CXCR5	PE	51505	Mouse [IgG2b, κ]	R&D Systems	10	4
PD1	APC	J105	Mouse [IgG1, κ]	eBioscience	20	4
CCR4	PE/Vio770	REA279	Human [IgG1]	Miltenyi	11	5
CD25	PE	M-A251	Mouse [IgG1, κ]	BD Biosciences	5	5
CD127	Alexa Fluor 647	HIL-7R-M21	Mouse [IgG1, κ]	BD Biosciences	5	5

Table E2: Antibody cell surface markers panels used in immunophenotyping flow cytometry. Immunophenotyping of PBMCs was performed in panels. FSC: Forward scatter; SSC: Side scatter; PD-1: Programmed cell death protein-1.

**Cell Population**	**FSC**	**SSC**	**Cell surface markers**							**Panel**
Myeloid										
Monocytes	Low/Mid	Low/Mid	CD3^{-}	CD19/20^{-}	CD14^{+}					1
Classical Monocyte	Low/Mid	Low/Mid	CD3^{-}	CD19/20^{-}	CD14^{+}	CD16^{-}				1
Non - Classical Monocyte	Low/Mid	Low/Mid	CD3^{-}	CD19/20^{-}	CD14^{+}	CD16^{+}				1
Natural killer	Low/Mid	Low/Mid	CD3^{-}	CD19/20^{-}	CD14^{-}	CD56^{+}				1
Dendritic cells (DCs)	Low/Mid	Low/Mid	CD3^{-}	CD19/20^{-}	CD14^{-}	CD56^{-}	HLA-DR^{+}			1
Plasmacytoid DCs	Low/Mid	Low/Mid	CD3^{-}	CD19/20^{-}	CD14^{-}	CD56^{-}	HLA-DR^{+}	CD123^{+}	CD11c^{-}	1
Myeloid DCs	Low/Mid	Low/Mid	CD3^{-}	CD19/20^{-}	CD14^{-}	CD56^{-}	HLA-DR^{+}	CD123^{-}	CD11c^{+}	1
Lymphocytes										
B cells										
B cells	Low	Low	CD3^{-}	CD19^{+}						2
Naïve B cells	Low	Low	CD3^{-}	CD19^{+}	IgD^{++}	CD27^{-}				2
Non-switched memory B cells	Low	Low	CD3^{-}	CD19^{+}	IgD^{+}	CD27^{+}				2
Transitional B cells	Low	Low	CD3^{-}	CD19^{+}	IgD^{++}	CD24^{+}	CD38^{+}			2
Plasmablasts	Low	Low	CD3^{-}	CD19^{+}	IgD^{-}	CD38^{++}				2
Switched memory B cells	Low	Low	CD3^{-}	CD19^{+}	IgD^{-}	CD38^{+}	CD27^{+}			2
Double negative B cells	Low	Low	CD3^{-}	CD19^{+}	IgD^{-}	CD38^{-}	CD27^{-}			2
Broad T cells										
CD3^{+} T cells	Low	Low	CD3^{+}							3
CD4^{+} T cells	Low	Low	CD3^{+}	CD4^{+}	CD8^{-}					3
CD8^{+} T cells	Low	Low	CD3^{+}	CD8^{+}	CD4^{-}					3
CD4^{+} Naïve	Low	Low	CD3^{+}	CD4^{+}	CD8^{-}	CD45RA^{+}	CCR7^{+}			3
CD4^{+} Effector memory	Low	Low	CD3^{+}	CD4^{+}	CD8^{-}	CD45RA^{-}	CCR7^{-}			3
CD4^{+} Effector	Low	Low	CD3^{+}	CD4^{+}	CD8^{-}	CD45RA^{+}	CCR7^{-}			3
CD4^{+} Central Memory	Low	Low	CD3^{+}	CD4^{+}	CD8^{-}	CD45RA^{-}	CCR7^{+}			3

$CD8^{+}$ Naïve	Low	Low	$CD3^{+}$	$CD8^{+}$	$CD4^{-}$	$CD45RA^{+}$	$CCR7^{+}$			3
$CD8^{+}$ Effector memory	Low	Low	$CD3^{+}$	$CD8^{+}$	$CD4^{-}$	$CD45RA^{-}$	$CCR7^{-}$			3
$CD8^{+}$ Effector	Low	Low	$CD3^{+}$	$CD8^{+}$	$CD4^{-}$	$CD45RA^{+}$	$CCR7^{-}$			3
$CD8^{+}$ Central memory	Low	Low	$CD3^{+}$	$CD8^{+}$	$CD4^{-}$	$CD45RA^{-}$	$CCR7^{+}$			3
T helper / follicular helper cells										4
T_H1	Low	Low	$CD3^{+}$	$CD4^{+}$	$CD8^{-}$	$CXCR3^{+}$	$CCR6^{-}$			4
T_H2	Low	Low	$CD3^{+}$	$CD4^{+}$	$CD8^{-}$	$CXCR3^{-}$	$CCR6^{-}$			4
T_H17	Low	Low	$CD3^{+}$	$CD4^{+}$	$CD8^{-}$	$CXCR3^{-}$	$CCR6^{+}$			4
$T_H1,17$	Low	Low	$CD3^{+}$	$CD4^{+}$	$CD8^{-}$	$CXCR3^{+}$	$CCR6^{+}$			4
$CD4^{+}$ $PD\text{-}1^{+}$	Low	Low	$CD3^{+}$	$CD4^{+}$	$CD8^{-}$	$PD\text{-}1^{+}$				4
$CD8^{+}$ $PD\text{-}1^{+}$	Low	Low	$CD3^{+}$	$CD8^{+}$	$CD4^{-}$	$PD\text{-}1^{+}$				4
Circulating T_{FH}	Low	Low	$CD3^{+}$	$CD4^{+}$	$CD8^{-}$	$CXCR5^{+}$	$CD45RA^{-}$	$PD\text{-}1^{+}$		4
$CD4^{+}$ $CXCR5^{+}$	Low	Low	$CD3^{+}$	$CD4^{+}$	$CXCR5^{+}$					4
$CD8^{+}$ $CXCR5^{+}$	Low	Low	$CD3^{+}$	$CD8^{+}$	$CXCR5^{+}$					4
Regulatory T cell										
T_{REG}	Low	Low	$CD3^{+}$	$CD4^{+}$	$CD25^{+}$	$CD127^{-}$				5
$CCR4^{+}$ T_{REG}	Low	Low	$CD3^{+}$	$CD4^{+}$	$CD25^{+}$	$CD127^{-}$	$CCR4^{+}$			5
Naïve T_{REG}	Low	Low	$CD3^{+}$	$CD4^{+}$	$CD25^{+}$	$CD127^{-}$	$CD45RA^{-}$			5
HLA-DR T_{REG}	Low	Low	$CD3^{+}$	$CD4^{+}$	$CD25^{+}$	$CD127^{-}$	$HLA\text{-}DR^{+}$			5

Supplementary Methods Figures – Legends

Figure E1: Overview of gating strategy used in immunophenotyping. Peripheral blood mononuclear cells (PBMCs) were first gated according to forward scatter (FSC) and side scatter (SSC) in order to identify cell populations. Cells were then gated for singlets and live cells (LIVE/DEAD). Antibody panels were used for the detection of sub-populations as follows: Broad T cell populations; regulatory T cell populations; helper and follicular helper T cells; B cells and myeloid cells. Boxed areas represent population subsets. HLA-DR: human leukocyte antigen – DR isotype; PD-1: programmed cell death protein – 1.

Figure E2: Assessment of optimal number of clusters for autoantibody clustering. A) Silhouette plot; B) Elbow plot of Log_e transformed autoantibody levels for PAM clustering with Euclidean distances. The silhouette plot and elbow plot indicate the optimal number of clusters (K) for PAM clustering with Euclidean distances. In A) the best performance is seen for K = 2, with K = 3 being a close second. In B) the first bend in the plot is seen at K = 3 with another at K = 5. The combination of these results suggests that K = 3 is the optimal choice for clustering, which fits with clinical expectations.

Figure E3: Development of a novel ELISA for the detection of autoantibodies to the BMPR2 extracellular domain (ECD). A) Serum from a healthy donor and a PAH patient demonstrating IgG reactivity was titrated to determine optimal sample dilution. B) Co-efficient of variation of two PAH samples shows good inter-plate variation, co-efficient of variation shown as % CV. C) Examples of test for non-specific binding of patient serum demonstrating IgG reactivity to the BMPR2 ECD. Samples were incubated both in the presence and absence of ECD and calculated as percentage binding. Sera with high levels of non-specific absorbance were removed.

Supplementary Results Tables

Supplementary Table E3: Frequencies of peripheral immune cell subsets from patients with IPAH and healthy donor controls as determined by flow cytometry. Data shown as percentage of parent gate, either mean ± standard deviation (*) with unpaired t-test or median [IQR 25,75%] with Mann Whitney non-parametric t-test (#) dependent on data normality as defined by D'Agostino & Pearson test for IPAH (n=26) and healthy donor controls (n=29). To control for multiple hypothesis testing, false discovery rates were estimated using Benjamini and Hochberg procedure on a per panel basis, and resultant q values are presented. We report here significant tests q<0.05. Lymphocyte, $CD3^+$ T cells, $CD4^+$ T cells and $CD8^+$ T cells averaged across multiple panels. DC: Dendritic cells; PD-1: programmed cell death protein-1; HLA-DR: Human leukocyte antigen-DR isotype.

Phenotypic Markers	Population	Healthy Control (%)	IPAH (%)	p-value	FDR adjusted q-value
**Myeloid panel**					
$CD3^-$, $CD19/20^-$, $CD14^+$	Monocytes*	50.31 (±15.30)	53.85 (±15.90)	0.409	0.617
$CD16^-$ Monocytes	Monocytes – Classical#	94.90 [89.28, 96.58]	94.65 [91.90, 97.05]	0.372	0.617
$CD16^+$ Monocyte	Monocytes – Non-classical#	4.27 [2.34, 9.10]	3.91 [2.00, 5.74]	0.441	0.617
$CD3^-$, $CD19/20^-$, $CD14^-$, $CD56^-$, $HLA\text{-}DR^+$	Dendritic cells*	26.52 (±12.84)	32.18 (±16.65)	0.165	0.577
$CD123^+$, $CD11c^-$ DC's	Plasmacytoid DC*	11.71 (±9.36)	7.51 (±5.12)	0.051	0.355

CD11c$^+$, CD123$^-$ DC's	Myeloid DC$^\#$	21.10 [18.73, 23.00]	20.00 [13.50, 34.85]	0.800	0.800
CD3$^-$, CD19/20$^-$, CD14$^-$, CD56$^+$	Natural Killer cells*	16.06 (±9.58)	18.02 (±13.66)	0.538	0.628
**Lymphocytes* (average)**		53.12 (±16.61)	40.46 (±17.15)	0.0075	0.09
**B cell panel**					
CD3$^-$, CD19$^+$	B cells$^\#$	2.01 [1.25, 3.27]	2.63 [1.64, 3.98]	0.093	0.108
CD27$^-$, IgD$^+$ B cells	Naïve B cells*	54.09 (±13.22)	61.21 (±14.98)	0.067	0.093
CD24$^+$, CD38$^+$, naïve B cells	Transitional B cells$^\#$	6.94 [5.65, 7.88]	5.89 [3.94, 10.47]	0.885	0.885
CD27$^+$, IgD$^+$ B cells	Non-switched memory B cells$^\#$	7.74 [5.49, 14.80]	5.29 [3.45, 7.16]	0.0065	0.0303
CD38$^+$, CD20$^-$, IgD$^-$ B cells	Plasmablasts$^\#$	4.69 [3.88, 6.90]	7.60 [5.47, 17.68]	0.017	0.0303
CD27$^+$, IgD$^-$ B cells	Switched memory B cells$^\#$	78.78 [74.50, 83.16]	73.03 [65.05, 79.03]	0.015	0.0303
CD38$^-$, CD27$^-$ IgD$^-$ B cells	Double Negative B cells$^\#$	21.23 [16.84, 25.50]	26.98 [20.98, 34.95]	0.015	0.0303
**T cell panel**					
**T cells (all averaged)**					
CD3$^+$	T Cells*	66.16 (±10.63)	59.80 (±12.97)	0.051	0.305
CD4$^+$ T cells	CD4+ T cells$^\#$	57.20 [47.65, 62.00]	54.05 [28.58, 54.05]	0.818	0.818
CD8$^+$ T cells	CD8+ T cells*	22.15 (±7.91)	23.39 (±8.26)	0.573	0.726

**$CD4^+$ subsets**					
$CD45RA^+$, $CCR7^+$, $CD4^+$ T cells	Naïve*	48.43 (±13.03)	46.44 (±16.60)	0.635	0.726
$CD45RA^-$, $CCR7^+$, $CD4^+$ T cells	Central Memory*	27.45 (±12.48)	26.06 (±10.04)	0.665	0.726
$CD45RA^-$, $CCR7^-$, $CD4^+$ T cells	Effector Memory*	18.95 (±6.30)	23.19 (±11.54)	0.108	0.430
$CD45RA^+$, $CCR7^-$, $CD4^+$ T cells	Effector[#]	1.89 [1.22, 2.77]	2.39 [1.15, 4.62]	0.474	0.712
**CD8+ subsets**					
$CD45RA^+$, $CCR7^+$, $CD8^+$ T cells	Naïve*	36.51 (±16.67)	32.74 (±18.79)	0.451	0.712
$CD45RA^-$, $CCR7^+$, $CD8^+$ T cells	Central Memory[#]	7.03 [4.08, 14.09]	5.39 [3.79, 8.23]	0.187	0.464
$CD45RA^-$, $CCR7^-$, $CD8^+$ T cells	Effector Memory*	33.40 (±15.32)	39.69 (±19.65)	0.207	0.464
$CD45RA^+$, $CCR7^-$, $CD8^+$ T cells	Effector[#]	10.80 [5.50, 23.88]	17.60 [10.44, 31.35]	0.232	0.464
**T Helper / Follicular helper panel**					
$CCR6^-$, $CXCR3^+$, $CD4^+$ T cells	T_H1*	16.98 (±9.67)	18.34 (±8.39)	0.584	0.702
$CCR6^-$, $CXCR3^-$, $CD4^+$ T cells	T_H2*	24.76 (±10.02)	29.73 (±10.94)	0.088	0.193
$CCR6^+$, $CXCR3^-$, $CD4^+$ T cells	T_H17*	41.66 (±13.21)	36.75 (±7.91)	0.107	0.193
$CCR6^+$, $CXCR3^+$, $CD4^+$ T cells	$T_H1,17$ [#]	15.40 [9.15, 23.00]	12.50 [9.93, 21.43]	0.683	0.702
**T follicular helper cells**					

PD1$^+$, CXCR5$^+$, CD45RA$^-$, CD4$^+$ T cells	Circulating T_{FH} #	3.82 [3.46, 4.73]	8.75 [4.43, 12.56]	<0.0001	0.0009
CXCR5$^+$, CD4$^+$, T cells	CXCR5$^+$ CD4$^+$ #	10.09 [6.04, 13.73]	9.51 [7.23, 11.51]	0.702	0.702
CXCR5$^+$, CD8$^+$ T cells	CXCR5$^+$ CD8$^+$ #	0.95 [0.56, 1.70]	0.80 [054, 1.44]	0.568	0.702
**PD1$^+$ T Cells**					
PD1$^+$ CD4$^+$ T cells	CD4$^+$ PD1$^+$ #	4.07 [2.95, 4.90]	4.79 [3.21, 8.46]	0.103	0.193
PD1$^+$ CD8$^+$ T cells	CD8$^+$ PD1$^+$ #	3.51 [3.15, 3.88]	4.57 [3.04, 7.22]	0.037	0.168
**Regulatory T cell panel**					
CD25$^+$, CD127$^-$, CD4$^+$ T cells	T_{REG}*	6.51 (±2.10)	8.57 (±3.73)	0.018	0.0402
CD45RA$^-$ T_{REG} cells	Naïve T_{REG}#	62.10 [53.80, 72.70]	66.20 [58.53, 71.73]	0.300	0.400
CCR4$^+$ T_{REG} cells	CCR4$^+$ Primed T_{REG}#	7.81 [7.01, 13.00]	5.53 [2.56, 10.37]	0.020	0.0402
HLA-DR$^+$ T_{REG} cells	HLA-DR Activated T_{REG}#	23.60 [15.20, 29.60]	24.48 [16.23, 31.88]	0.546	0.546

Supplementary Table E4. Clinical characteristics of PAH and healthy donor control subjects analysed for autoimmunity autoantibody biomarkers. Numeric data reported as mean ± standard deviation and count data are reported as count (percentage per group). Differences between groups were assessed with chi-square tests and ANOVAs for the PAH subtypes and chi-square tests and independent T-test for case status. To control for multiple hypothesis testing, false discovery rates were estimated using the Benjamini and Hochberg procedure within each set of 22 tests, and resultant q values are presented. We report here as significant tests $q<0.05$. Additional comorbid autoimmune diseases assessed for but not detected: SLE, systemic sclerosis, undifferentiated connective tissue disease, necrotising vasculopathies and overlap syndrome.

Supplementary Table E4

		HPAH (n=47)	IPAH (n=415)	PCH (n=1)	PVOD (n=10)	Total (n=473)	*p*-value	FDR-adjusted q-value	Incident (n=135)	Prevalent (n=334)	Total (n=469)	p-value	FDR-adjusted *q*-value
**Demographics**													
Sex	Female	33 (70.2%)	292 (70.4%)	0 (0.0%)	7 (70.0%)	332 (70.2%)	0.50	0.99	81 (60.0%)	248 (74.3%)	329 (70.1%)	0.0033	0.018
	Male	14 (29.8%)	123 (29.6%)	1 (100.0%)	3 (30.0%)	141 (29.8%)			54 (40.0%)	86 (25.7%)	140 (29.9%)		
Case status*	Incident	14 (29.8%)	110 (26.8%)	1 (100.0%)	10 (100.0%)	135 (28.8%)	3.53e-6	2.47e-5					
	Prevalent	33 (70.2%)	301 (73.2%)	0 (0.0%)	0 (0.0%)	334 (71.2%)							
PAH status	HPAH								14 (10.4%)	33 (9.9%)	47 (10.0%)	3.53e-6	3.71e-5
	IPAH								110 (81.5%)	301 (90.1%)	411 (87.6%)		
	PCH								1 (0.7%)	0 (0.0%)	1 (0.2%)		
	PVOD								10 (7.4%)	0 (0.0%)	10 (2.1%)		
**Haemodynamic parameters**													
PAWP* (mmHg)		8.7 (±3.6)	9.3 (±3.9)	6	10.3 (±2.5)	9.3 (3±.8)	0.52	0.99	8.6 (±3.3)	9.6 (±4.0)	9.3 (±3.8)	0.013	0.055
PVR* (Wood Units)		15.7 (±7.0)	11.9 (±5.6)	5	10 (±3.8)	12.2 (±5.8)	3.3e-4	0.0017	12 (±5.5)	12.3 (±5.9)	12.2 (±5.8)	0.63	0.75
mPAP* (mmHg)		56.5 (±13)	53 (±13.1)	25	48.8 (±12.2)	53.2 (±13.1)	0.032	0.13	50.4 (±12.6)	54.3 (±13.2)	53.2 (±13.1)	0.0040	0.018
**Risk scores and functional status**													
REVEAL risk score*	Very high risk	6 (12.8%)	8 (1.9%)	0 (0.0%)	6 (60.0%)	20 (4.2%)	2.20e-16	4.62e-15	12 (8.9%)	8 (2.4%)	20 (4.3%)	3.56e-7	7.47e-6
	High risk	12 (25.5%)	51 (12.3%)	0 (0.0%)	2 (20.0%)	65 (13.7%)			28 (20.7%)	37 (11.1%)	65 (13.9%)		
	Moderate risk	14 (29.8%)	51 (12.3%)	0 (0.0%)	2 (20.0%)	67 (14.2%)			31 (23.0%)	36 (10.8%)	67 (14.3%)		
	Low risk	5 (10.6%)	197 (47.5%)	1 (100.0%)	0 (0.0%)	203 (42.9%)			39 (28.9%)	164 (49.1%)	203 (43.3%)		
WHO functional class*	1	0 (0.0%)	7 (1.7%)	1 (100.0%)	0 (0.0%)	8 (1.7%)	2.12e-10	2.23e-9	2 (1.5%)	6 (1.8%)	8 (1.7%)	0.033	0.12
	2	9 (19.1%)	74 (17.8%)	0 (0.0%)	0 (0.0%)	83 (17.5%)			14 (10.4%)	69 (20.7%)	83 (17.7%)		
	3	32 (68.1%)	263 (63.4%)	0 (0.0%)	6 (60.0%)	301 (63.6%)			96 (71.1%)	205 (61.4%)	301 (64.2%)		
	4	5 (10.6%)	51 (12.3%)	0 (0.0%)	4 (40.0%)	60 (12.7%)			22 (16.3%)	38 (11.4%)	60 (12.8%)		
**Clinical indications of autoimmunity**													
Comorbid hypothyroidism	No	41 (87.2%)	355 (85.5%)	1 (100.0%)	9 (90.0%)	406 (85.8%)	0.94	0.99	121 (89.6%)	281 (84.1%)	402 (85.7%)	0.16	0.36
	Yes	6 (12.8%)	60 (14.5%)	0 (0.0%)	1 (10.0%)	67 (14.2%)			14 (10.4%)	53 (15.9%)	67 (14.3%)		

Comorbid diabetes mellitus type 1	No	47 (100.0%)	408 (98.3%)	1 (100.0%)	10 (100.0%)	466 (98.5%)	0.8029	0.9876	133 (98.5%)	329 (98.5%)	462 (98.5%)	1.00	1.00
	Yes	0 (0.0%)	7 (1.7%)	0 (0.0%)	0 (0.0%)	7 (1.5%)			2 (1.5%)	5 (1.5%)	7 (1.5%)		
Comorbid Sjögren's	No	46 (97.9%)	414 (99.8%)	1 (100.0%)	10 (100.0%)	471 (99.6%)	0.31	0.92	135 (100.0%)	332 (99.4%)	467 (99.6%)	0.91	0.95
	Yes	1 (2.1%)	1 (0.2%)	0 (0.0%)	0 (0.0%)	2 (0.4%)			0 (0.0%)	2 (0.6%)	2 (0.4%)		
Comorbid ankylosing spondylitis	No	47 (100.0%)	414 (99.8%)	1 (100.0%)	10 (100.0%)	472 (99.8%)	0.99	0.99	134 (99.3%)	334 (100.0%)	468 (99.8%)	0.64	0.75
	Yes	0 (0.0%)	1 (0.2%)	0 (0.0%)	0 (0.0%)	1 (0.2%)			1 (0.7%)	0 (0.0%)	1 (0.2%)		
Comorbid polymyalgia rheumatica	No	47 (100.0%)	414 (99.8%)	1 (100.0%)	10 (100.0%)	472 (99.8%)	0.99	0.99	134 (99.3%)	334 (100.0%)	468 (99.8%)	0.64	0.75
	Yes	0 (0.0%)	1 (0.2%)	0 (0.0%)	0 (0.0%)	1 (0.2%)			1 (0.7%)	0 (0.0%)	1 (0.2%)		
Clinical autoimmunity suspicion/evidence*	No	26 (55.3%)	239 (57.6%)	1 (100.0%)	6 (60.0%)	272 (57.5%)	0.81	0.99	99 (73.3%)	173 (51.8%)	272 (58.0%)	0.42	0.64
	Yes	6 (12.8%)	43 (10.4%)	0 (0.0%)	2 (20.0%)	51 (10.8%)			15 (11.1%)	36 (10.8%)	51 (10.9%)		
**Autoantibody status**													
ANA*	Negative	30 (81.1%)	260 (82.8%)	1 (100.0%)	7 (87.5%)	298 (82.8%)	0.99	0.99	97 (84.3%)	201 (82.0%)	298 (82.8%)	0.83	0.91
	Positive	4 (10.8%)	31 (9.9%)	0 (0.0%)	1 (12.5%)	36 (10.0%)			11 (9.6%)	25 (10.2%)	36 (10.0%)		
Anti-cardiolipin*	Negative	16 (45.7%)	141 (45.8%)	1 (100.0%)	2 (28.6%)	160 (45.6%)	0.83	0.99	60 (52.6%)	100 (42.2%)	160 (45.6%)	0.17	0.36
	Positive	0 (0.0%)	5 (1.6%)	0 (0.0%)	0 (0.0%)	5 (1.4%)			1 (0.9%)	4 (1.7%)	5 (1.4%)		
Anti-dsDNA*	Negative	21 (58.3%)	148 (50.0%)	0 (0.0%)	2 (25.0%)	171 (50.1%)	0.61	0.99	51 (47.2%)	120 (51.5%)	171 (50.1%)	0.35	0.58
	Positive	0 (0.0%)	3 (1.0%)	0 (0.0%)	0 (0.0%)	3 (0.9%)			2 (1.9%)	1 (0.4%)	3 (0.9%)		
Anti-SCL-70*	Negative	12 (32.4%)	129 (41.0%)	1 (100.0%)	3 (37.5%)	145 (40.2%)	0.85	0.99	51 (44.0%)	94 (38.4%)	145 (40.2%)	0.49	0.68
	Positive	0 (0.0%)	1 (0.3%)	0 (0.0%)	0 (0.0%)	1 (0.3%)			0 (0.0%)	1 (0.4%)	1 (0.3%)		
Anti-centromere*	Negative	15 (40.5%)	136 (43.0%)	1 (100.0%)	2 (25.0%)	154 (42.5%)	0.85	0.99	55 (47.4%)	99 (40.2%)	154 (42.5%)	0.36	0.58
	Positive	0 (0.0%)	1 (0.3%)	0 (0.0%)	0 (0.0%)	1 (0.3%)			0 (0.0%)	1 (0.4%)	1 (0.3%)		
Anti-Rho*	Negative	11 (30.6%)	117 (37.0%)	1 (100.0%)	3 (37.5%)	132 (36.6%)	0.79	0.99	49 (42.6%)	83 (33.7%)	132 (36.6%)	0.24	0.46
	Positive	0 (0.0%)	5 (1.6%)	0 (0.0%)	0 (0.0%)	5 (1.4%)			1 (0.9%)	4 (1.6%)	5 (1.4%)		
Anti-ENA*	Negative	21 (56.8%)	183 (58.1%)	1 (100.0%)	2 (25.0%)	207 (57.3%)	0.41	0.99	57 (49.1%)	150 (61.2%)	207 (57.3%)	0.094	0.25
	Positive	1 (2.7%)	2 (0.6%)	0 (0.0%)	0 (0.0%)	3 (0.8%)			1 (0.9%)	2 (0.8%)	3 (0.8%)		
ANCA*	Negative	23 (62.2%)	149 (47.6%)	0 (0.0%)	2 (28.6%)	174 (48.6%)	0.30	0.92	46 (40.7%)	128 (52.2%)	174 (48.6%)	0.087	0.25
	Positive	1 (2.7%)	10 (3.2%)	0 (0.0%)	1 (14.3%)	12 (3.4%)			3 (2.7%)	9 (3.7%)	12 (34%)		

*Missing / not available data: Case status n = 4; PAWP n= 71; PVR n = 77; mPAP n = 21; REVEAL risk n = 118; WHO classification n = 21; Clinical autoimmunity evidence / suspicion n = 150; Annti-nuclear antibody (ANA): n =150; Anti-cardiolipin n = 308; Anti-dsDNA n = 299; Anti-SCL70 n = 327; Anti-centromere n = 318; Anti-Rho n = 336; Anti-Extractable nuclear antigen (ENA) n = 263; Anti-neutrophil cytoplasmic antibodies (ANCA) n = 287

Supplementary Table E5: Differences in autoantibody positivity between cases and controls. Autoantibody positivity was defined as 0.75Q + 2IQR of the control population. Differences in positivity ratio were compared using a Chi-square test. To control for multiple hypothesis testing, FDR-adjusted q-values were calculated across 19 tests using the Benjamini and Hochberg procedure, and resultant q values are presented. We report here as significant tests q<0.05. Data shown as counts (percentage per group).

Autoantibody	Autoantibody Positivity	Healthy controls (n=946)	PAH patients (n=473)	Total (n=1419)	FDR-adjusted q-value
Cardiolipin	Negative	853 (90.2%)	366 (77.4%)	1219 (85.9%)	< 1e-04
	Positive	93 (9.8%)	107 (22.6%)	200 (14.1%)	
Centromere protein B	Negative	877 (92.7%)	444 (93.9%)	1321 (93.1%)	0.65
	Positive	69 (7.3%)	29 (6.1%)	98 (6.9%)	
H2a (F2a2) & H4 (F2a1)	Negative	870 (92.0%)	408 (86.3%)	1278 (90.1%)	0.0027
	Positive	76 (8.0%)	65 (13.7%)	141 (9.9%)	
Histone type IIa	Negative	868 (91.8%)	439 (92.8%)	1307 (92.1%)	0.70
	Positive	78 (8.2%)	34 (7.2%)	112 (7.9%)	
Jo-1	Negative	913 (96.5%)	448 (94.7%)	1361 (95.9%)	0.22
	Positive	33 (3.5%)	25 (5.3%)	58 (4.1%)	
Mi-2b	Negative	885 (93.6%)	433 (91.5%)	1318 (92.9%)	0.29
	Positive	61 (6.4%)	40 (8.5%)	101 (7.1%)	
La/SS-B	Negative	836 (88.4%)	352 (74.4%)	1188 (83.7%)	< 1e-04
	Positive	110 (11.6%)	121 (25.6%)	231 (16.3%)	
Myeloperoxidase	Negative	874 (92.4%)	433 (91.5%)	1307 (92.1%)	0.74
	Positive	72 (7.6%)	40 (8.5%)	112 (7.9%)	
Proteinase-3	Negative	894 (94.5%)	373 (78.9%)	1267 (89.3%)	< 1e-04
	Positive	52 (5.5%)	100 (21.1%)	152 (10.7%)	
Pyruvate dehydrogenase	Negative	869 (91.9%)	418 (88.4%)	1287 (90.7%)	0.072
	Positive	77 (8.1%)	55 (11.6%)	132 (9.3%)	
RNP-complex	Negative	855 (90.4%)	372 (78.6%)	1227 (86.5%)	< 1e-04
	Positive	91 (9.6%)	101 (21.4%)	192 (13.5%)	
Ro/SS-A antigen	Negative	864 (91.3%)	454 (96.0%)	1318 (92.9%)	0.0040
	Positive	82 (8.7%)	19 (4.0%)	101 (7.1%)	
SCL-70 antigen	Negative	856 (90.5%)	424 (89.6%)	1280 (90.2%)	0.74
	Positive	90 (9.5%)	49 (10.4%)	139 (9.8%)	
Scl-34	Negative	841 (88.9%)	417 (88.2%)	1258 (88.7%)	0.74
	Positive	105 (11.1%)	56 (11.8%)	161 (11.3%)	
Smith antigen	Negative	863 (91.2%)	386 (81.6%)	1249 (88.0%)	< 1e-04
	Positive	83 (8.8%)	87 (18.4%)	170 (12.0%)	
Thyroglobulin	Negative	891 (94.2%)	422 (89.2%)	1313 (92.5%)	0.0028
	Positive	55 (5.8%)	51 (10.8%)	106 (7.5%)	

Thyroid peroxidase	Negative	762 (80.5%)	349 (73.8%)	1111 (78.3%)	0.0084
	Positive	184 (19.5%)	124 (26.2%)	308 (21.7%)	
Transglutaminase	Negative	896 (94.7%)	445 (94.1%)	1341 (94.5%)	0.74
	Positive	50 (5.3%)	28 (5.9%)	78 (5.5%)	
u1-snRNP 68	Negative	893 (94.4%)	408 (86.3%)	1301 (91.7%)	< 1e-04
	Positive	53 (5.6%)	65 (13.7%)	118 (8.3%)	

Supplementary Table E6: Differences in autoantibody positivity in PAH patients between clusters. Stratification of autoantibody positivity between clustered groups. PAM with K=3 was defined as optimal after clustering and differences in prevalence of statistical autoantibody positivity were assessed with a Chi-square test. To control for multiple hypothesis testing, FDR q-values were calculated across 19 tests using the Benjamini and Hochberg procedure, Resultant q values are presented. We report here as significant tests q<0.05.

Autoantibody	Positivity	High autoantibody cluster (n=130)	Low autoantibody cluster (n=290)	Intermediate autoantibody cluster (n=53)	Total (n=473)	FDR-adjusted q-value
Cardiolipin	Negative	46 (35.4%)	279 (96.2%)	41 (77.4%)	366 (77.4%)	0.00079
	Positive	84 (64.6%)	11 (3.8%)	12 (22.6%)	107 (22.6%)	
CENP-B	Negative	116 (89.2%)	280 (96.6%)	48 (90.6%)	444 (93.9%)	0.011
	Positive	14 (10.8%)	10 (3.4%)	5 (9.4%)	29 (6.1%)	
H2a & H4	Negative	78 (60.0%)	288 (99.3%)	42 (79.2%)	408 (86.3%)	0.00079
	Positive	52 (40.0%)	2 (0.7%)	11 (20.8%)	65 (13.7%)	
Histone-IIa	Negative	106 (81.5%)	287 (99.0%)	46 (86.8%)	439 (92.8%)	0.00079
	Positive	24 (18.5%)	3 (1.0%)	7 (13.2%)	34 (7.2%)	
Jo-1	Negative	115 (88.5%)	285 (98.3%)	48 (90.6%)	448 (94.7%)	0.00079
	Positive	15 (11.5%)	5 (1.7%)	5 (9.4%)	25 (5.3%)	
Mi-2b	Negative	114 (87.7%)	271 (93.4%)	48 (90.6%)	433 (91.5%)	0.15
	Positive	16 (12.3%)	19 (6.6%)	5 (9.4%)	40 (8.5%)	
La/SS-B	Negative	42 (32.3%)	268 (92.4%)	42 (79.2%)	352 (74.4%)	0.00079
	Positive	88 (67.7%)	22 (7.6%)	11 (20.8%)	121 (25.6%)	
Myeloperoxidase	Negative	109 (83.8%)	277 (95.5%)	47 (88.7%)	433 (91.5%)	0.0015
	Positive	21 (16.2%)	13 (4.5%)	6 (11.3%)	40 (8.5%)	
Proteinase-3	Negative	45 (34.6%)	285 (98.3%)	43 (81.1%)	373 (78.9%)	0.00079
	Positive	85 (65.4%)	5 (1.7%)	10 (18.9%)	100 (21.1%)	
Pyruvate dehydrogenase	Negative	107 (82.3%)	265 (91.4%)	46 (86.8%)	418 (88.4%)	0.030
	Positive	23 (17.7%)	25 (8.6%)	7 (13.2%)	55 (11.6%)	
RNP-complex	Negative	95 (73.1%)	277 (95.5%)	0 (0.0%)	372 (78.6%)	0.00079
	Positive	35 (26.9%)	13 (4.5%)	53 (100.0%)	101 (21.4%)	
Ro/SS-A	Negative	120 (92.3%)	286 (98.6%)	48 (90.6%)	454 (96.0%)	0.0027
	Positive	10 (7.7%)	4 (1.4%)	5 (9.4%)	19 (4.0%)	
SCL-70	Negative	113 (86.9%)	262 (90.3%)	49 (92.5%)	424 (89.6%)	0.47
	Positive	17 (13.1%)	28 (9.7%)	4 (7.5%)	49 (10.4%)	
Scl-34	Negative	117 (90.0%)	252 (86.9%)	48 (90.6%)	417 (88.2%)	0.59
	Positive	13 (10.0%)	38 (13.1%)	5 (9.4%)	56 (11.8%)	

Smith antigen	Negative	81 (62.3%)	267 (92.1%)	38 (71.7%)	386 (81.6%)	0.00079
	Positive	49 (37.7%)	23 (7.9%)	15 (28.3%)	87 (18.4%)	
Thyroglobulin	Negative	85 (65.4%)	290 (100.0%)	47 (88.7%)	422 (89.2%)	0.00079
	Positive	45 (34.6%)	0 (0.0%)	6 (11.3%)	51 (10.8%)	
Thyroid peroxidase	Negative	33 (25.4%)	276 (95.2%)	40 (75.5%)	349 (73.8%)	0.00079
	Positive	97 (74.6%)	14 (4.8%)	13 (24.5%)	124 (26.2%)	
Transglutaminase	Negative	110 (84.6%)	284 (97.9%)	51 (96.2%)	445 (94.1%)	0.00079
	Positive	20 (15.4%)	6 (2.1%)	2 (3.8%)	28 (5.9%)	
u1-snRNP	Negative	79 (60.8%)	285 (98.3%)	44 (83.0%)	408 (86.3%)	0.00079
	Positive	51 (39.2%)	5 (1.7%)	9 (17.0%)	65 (13.7%)	

Supplementary Table E7. Demographic and clinical characteristics of autoantibody cluster analysis in PAH patients. Numeric data shown as mean ± standard deviation unless otherwise stated. Count data is represented as the count (percentage of total). Statistical analysis was performed using Chi-square tests and ANOVAs. To control for multiple hypothesis testing, FDR adjusted q-values were calculated across 178 tests using the Benjamini and Hochberg procedure, and resultant q values are presented. We report here as significant tests q<0.05. Significant FDR corrected numeric tests were subsequently subjected by ANCOVA analysis and corrected for sex, aetiology, age at diagnosis and BMI followed by Bonferroni correction for three tests. Log_e TSH levels were used for statistical analysis however numeric values are stated for observed levels. A mutation was defined as a minor allele frequency < 1:10,000 in one of the following genes: *KDR, BMPR2, EIF2AK4, SMAD4, ACVRL1, AQP1, ATP13A3, SMAD1, SMAD9, ENG, GDF2, KCNK3, SOX17*, and *TBX4*.

	**High autoantibody cluster (n=130)**	**Low autoantibody cluster (n=290)**	**Intermediate autoantibody cluster (n=53)**	**Total (n=473)**	**p-value**	**FDR-adjusted q-value**	**Bonferroni (For ANCOVA)**
Age at diagnosis*	44.3 ±15.3	48.9 ±17.1	49 ±15.9	47.6 ±16.6	0.028	0.13	
Age at sampling	50.8 ±14.5	53.8 ± 16.2	54.8 ± 14.8	53 ±15.6	0.14	0.32	
Sex							
Male	31 (23.8%)	95 (32.8%)	15 (28.3%)	141 (29.8%)	0.18	0.37	
Female	99 (76.2%)	195 (67.2%)	38 (71.7%)	332 (70.2%)			
BMI ± kg/m2*	27.8 ±6.3	28.9 ±7.1	30.7 ±7.6	28.8 ±7	0.043	0.17	
**Diagnosis**							
HPAH	10 (7.7%)	32 (11.0%)	5 (9.4%)	47 (9.9%)	0.70	0.83	
IPAH	118 (90.8%)	249 (85.9%)	48 (90.6%)	415 (87.7%)			
PVOD	0 (0.0%)	1 (0.3%)	0 (0.0%)	1 (0.2%)			
PCH	2 (1.5%)	8 (2.8%)	0 (0.0%)	10 (2.1%)			
**Mutations***							
Total PAH relevant mutation frequency	24 (18.5%)	82 (28.3%)	9 (17.0%)	115 (24.3%)	0.014	0.087	
BMPR2 mutation frequency	18 (13.8%)	53 (18.3%)	5 (9.4%)	76 (16.1%)	0.11	0.28	
**Haemodynamics**							
mPAP ± mmHg*	54.5 (12.9)	52.9 (13.4)	51.9 (12.1)	53.2 (13.1)	0.39	0.59	
mPAWP ± mmHg*	8.5 (4)	9.7 (3.6)	9.4 (4.1)	9.3 (3.8)	0.028	0.13	

mRAP ± mmHg*	9 (5.6)	9.1 (5.1)	8.5 (5.3)	9 (5.2)	0.73	0.83	
Cardiac output ± l/min *	3.7 (1.3)	4.1 (1.3)	4.4 (1.5)	4 (1.4)	0.0024	0.018	0.0084
Cardiac index*	2.1 (0.7)	2.2 (0.7)	2.4 (0.8)	2.2 (0.7)	0.022	0.12	
TSH ± mu/l*	3.5 (3.7)	2.1 (1.5)	2.8 (2.9)	2.6 (2.5)	0.066	0.20	
PVR ± Wood units*	14 (6.5)	11.7 (5.4)	10.8 (5.1)	12.2 (5.8)	0.00075	0.0063	0.0051
PVR-calc ± dyness-secs/cm^5 *	1119.4 (528)	941.4 (437.7)	858.2 (445)	980.9 (471.9)	0.00067	0.0060	0.022
6MWD distance ± meters*	341.4 (161)	314.8 (155.6)	306.4 (154.4)	321.1 (157.1)	0.25	0.51	
spO_2 pre-6MWD *	95.7 (3.1)	94.5 (4.1)	94.6 (4.1)	94.9 (3.9)	0.026	0.12	
spO_2 post-6MWD	91.9 (7.3)	89.4 (8.4)	88.1 (9.3)	90 (8.3)	0.013	0.083	
**WHO functional class***					0.0059	0.042	
1	4 (3.1%)	3 (1.0%)	1 (1.9%)	8 (1.7%)			
2	23 (17.7%)	48 (16.6%)	12 (22.6%)	83 (17.5%)			
3	67 (51.5%)	196 (67.6%)	38 (71.7%)	301 (63.6%)			
4	26 (20.0%)	33 (11.3%)	1 (1.9%)	60 (12.7%)			
**REVEAL Risk Score***					0.092	0.25	
Low	55 (42.3%)	119 (41.0%)	29 (54.7%)	203 (42.9%)			
Moderate	10 (7.7%)	51 (17.6%)	6 (11.3%)	67 (14.2%)			
High	20 (15.4%)	42 (14.5%)	3 (5.7%)	65 (13.7%)			
Very high	5 (3.8%)	13 (4.5%)	2 (3.8%)	20 (4.2%)			
**Indications of autoimmune disease**							
Co-morbid hypothyroidism					4.5e-6	4.2e-5	
Yes	33 (25.4%)	23 (7.9%)	11 (20.8%)	67 (14.2%)			
No	97 (74.6%)	267 (92.1%)	42 (79.2%)	406 (85.8%)			
Diabetes Mellitus type 1					0.63	0.77	
Yes	2 (1.5%)	5 (1.7%)	0 (0.0%)	7 (1.5%)			
No	128 (98.5%)	285 (98.3%)	53 (100.0%)	466 (98.5%)			
Sjögren's					0.071	0.20	
Yes	2 (1.5%)	0 (0.0%)	0 (0.0%)	2 (0.4%)			
No	128 (98.5%)	290 (100.0%)	53 (100.0%)	471 (99.6%)			
Ankylosing Spondylitis					0.73	0.83	
Yes	0 (0.0%)	1 (0.3%)	0 (0.0%)	1 (0.2%)			
No	130 (100.0%)	289 (99.7%)	53 (100.0%)	472 (99.8%)			
Polymyalgia rheumatica					0.73	0.83	
Yes	0 (0.0%)	1 (0.3%)	0 (0.0%)	1 (0.2%)			
No	130 (100.0%)	289 (99.7%)	53 (100.0%)	472 (99.8%)			
Overlap syndrome					0.73	0.83	
Yes	0 (0.0%)	1 (0.3%)	0 (0.0%)	1 (0.2%)			
No	130 (100.0%)	289 (99.7%)	53 (100.0%)	472 (99.8%)			

Clinical autoimmunity suspicion / evidence*					0.12	0.28	
Yes	16 (12.3%)	26 (9.0%)	9 (17.0%)	51 (10.8%)			
No	71 (54.6%)	175 (60.3%)	26 (49.1%)	272 (57.5%)			
**Autoantibodies**							
ANA*					0.16	0.36	
Positive	12 (9.2%)	17 (5.9%)	7 (13.2%)	36 (7.6%)			
Negative	79 (60.7%)	186 (64.1%)	33 (62.3%)	298 (63.0%)			
ANCA*					0.83	0.89	
Positive	3 (2.3%)	8 (2.8%)	1 (1.9%)	12 (2.5%)			
Negative	47 (36.2%)	103 (35.5%)	24 (45.3%)	174 (36.8%)			
Anti-Cardiolipin*					0.24	0.49	
Positive	3 (2.3%)	2 (0.7%)	0 (0.0%)	5 (1.1%)			
Negative	43 (33.1%)	98 (33.8%)	19 (35.8%)	160 (33.8%)			
Anti-dsDNA*					0.018	0.10	
Positive	3 (2.3%)	0 (0.0%)	0 (0.0%)	3 (0.6%)			
Negative	45 (34.6%)	105 (36.2%)	21 (39.6%)	171 (36.2%)			
Anti-SCL70*					0.38	0.59	
Positive	1 (0.8%)	0 (0.0%)	0 (0.0%)	1 (0.2%)			
Negative	49 (37.7%)	82 (28.3%)	14 (26.4%)	145 (30.7%)			
Anti-centromere*					0.013	0.083	
Positive	0 (0.0%)	0 (0.0%)	1 (1.9%)	1 (0.2%)			
Negative	44 (33.8%)	95 (32.8%)	15 (28.3%)	154 (32.6%)			
Anti-Rho*					0.088	0.24	
Positive	4 (3.1%)	1 (0.3%)	0 (0.0%)	5 (1.1%)			
Negative	43 (33.1%)	77 (26.6%)	12 (22.6%)	132 (27.9%)			
Anti-ENA*					0.28	0.52	
Positive	2 (1.5%)	1 (0.3%)	0 (0.0%)	3 (0.6%)			
Negative	54 (41.5%)	128 (44.1%)	25 (47.2%)	207 (43.8%)			
**Other relevant comorbidities**							
Type 2 diabetes mellitus					0.0039	0.029	
Yes	7 (5.4%)	46 (15.9%)	11 (20.8%)	64 (13.5%)			
No	123 (94.6%)	244 (84.1%)	42 (79.2%)	409 (86.5%)			
**Treatment**							
Type of treatment					0.026	0.12	
No medication recorded	5 (3.8%)	13 (4.5%)	0 (0.0%)	18 (3.8%)			
Single therapy	15 (11.5%)	33 (11.4%)	6 (11.3%)	54 (11.4%)			
Combination therapy	23 (17.7%)	82 (28.3%)	20 (37.7%)	125 (26.4%)			
Triple therapy	54 (41.5%)	123 (42.4%)	20 (37.7%)	197 (41.6%)			
Intravenous medication	33 (25.4%)	39 (13.4%)	7 (13.2%)	79 (16.7%)			

*Missing data: age at diagnosis n=4, BMI n=24, BMPR2 mutation frequency = 74, mPAP n=21, mPAWP n=73, mRAP n=41, cardiac output n=37, cardiac index n=47, TSH n=138, PVR n=77, PVR-calc n=61, 6MWD n=50, spO_2 pre 6MWD n=65, WHO functional class n=21, REVEAL risk score n= 118, Clinical autoimmunity suspicion / evidence n=150, ANA n=139, ANCA n=287, anti-cardiolipin n=308, anti-dsDNA n=299, anti-SCL70 n=327, anti-centromere n=318, anti-Rho n=336, anti-ENA n=263

BMI (Body Mass Index); mPAP (mean Pulmonary Arterial Pressure); mRAP (mean Right Atrial Pressure); TSH (Thyroid Stimulating Hormone); PVR (Pulmonary Vascular Resistance); PVR-calc (calculated PVR); 6MWD (six minutes walking distance test); spO_2 (oxygen saturation); ANA (anti-nuclear antibodies); ANCA (anti-neutrophil cytoplasmic antibodies); anti-ENA (anti – extractable nuclear antigen).

Supplementary Results Figures - Legends

Supplementary Figure E4: Correlation analysis of lymphocytes; $CD3^+$; $CD4^+$ T cell and $CD8^+$ T cell populations measured across different immunophenotyping panels. Samples from individuals were measured across multiple antibody panels and show good correlation. P values and r or r^2 were derived by Pearson or Spearman's rank correlation determined by normality of data.

Supplementary Figure E5: Correlations of leukocyte cell populations in IPAH patients. A) T_{REG} vs. Plasmablast; B) T_{REG} vs. circulating T_{FH}; C) Non-switched memory B cells vs. Plasmablasts. P values and r calculations derived from Pearson or Spearmans rank correlation depending on normality of data.

Supplementary Figure E6: Cox-proportional hazard model of survival differences between clusters. Survival differences between clusters were corrected for age at diagnosis, sex and treatment using a cox-proportional hazard model. Wald tests were used to determine p-values.

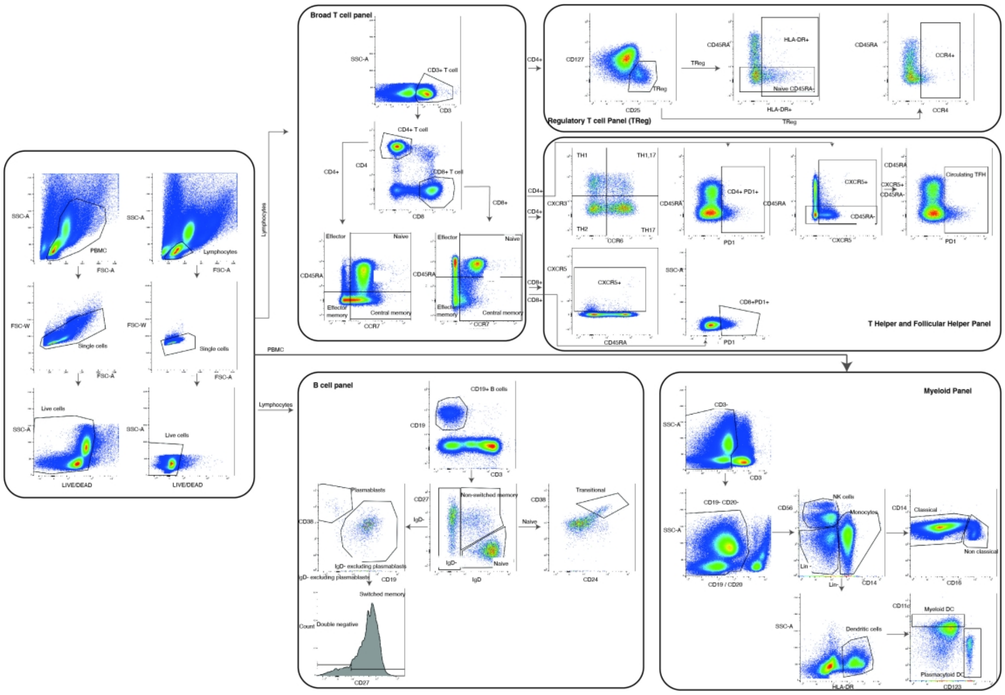

Figure E1: Overview of gating strategy used in immunophenotyping.
Peripheral blood mononuclear cells (PBMCs) were first gated according to forward scatter (FSC) and side scatter (SSC) in order to identify cell populations. Cells were then gated for singlets and live cells (LIVE/DEAD). Antibody panels were used for the detection of sub-populations as follows: Broad T cell populations; regulatory T cell populations; helper and follicular helper T cells; B cells and myeloid cells. Boxed areas represent population subsets. HLA-DR: human leukocyte antigen – DR isotype; PD-1: programmed cell death protein – 1.

286x197mm (300 x 300 DPI)

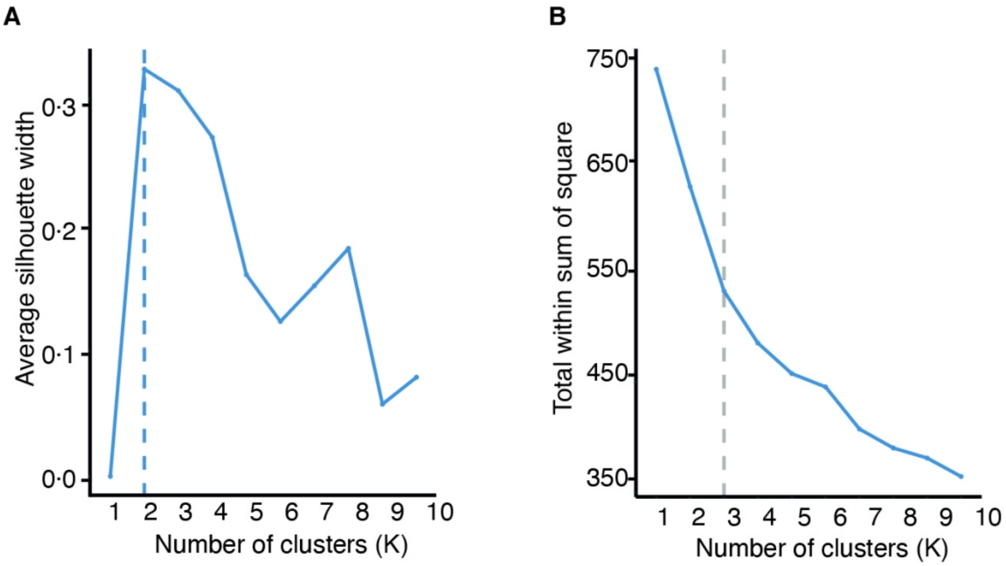

Figure E2: Assessment of optimal number of clusters for autoantibody clustering.
A) Silhouette plot; B) Elbow plot of Loge transformed autoantibody levels for PAM clustering with Euclidean distances. The silhouette plot and elbow plot indicate the optimal number of clusters (K) for PAM clustering with Euclidean distances. In A) the best performance is seen for K = 2, with K = 3 being a close second. In B) the first bend in the plot is seen at K = 3 with another at K = 5. The combination of these results suggests that K = 3 is the optimal choice for clustering, which fits with clinical expectations.

111x62mm (300 x 300 DPI)

A

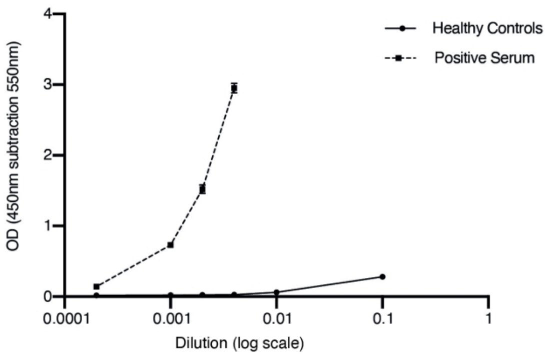

B

Sample	Mean OD	S.D.	% C.V.
A	0·67	0·11	15·9
B	0·16	0·011	7·3

C

Sample	Absorbance with ECD (450nm, 550nm subtrarction)	Absorbance without ECD (450nm, 550nm subtrarction)	Non-specific binding (%)
Sample A	1·51	0·11	7·34
Sample B	0·42	0·019	4·42
Sample C	1·15	0·019	1·61
Sample D	0·83	0·019	2·35
Sample E	0·19	0·016	8·36
Below samples removed due to high non-specific absorbance			
Sample F	2·01	0·63	31·15
Sample G	0·96	0·73	76·40
Sample H	1·11	1·26	114·20

Figure E3: Development of a novel ELISA for the detection of autoantibodies to the BMPR2 extracellular domain (ECD). A) Serum from a healthy donor and a PAH patient demonstrating IgG reactivity was titrated to determine optimal sample dilution. B) Co-efficient of variation of two PAH samples shows good inter-plate variation, co-efficient of variation shown as % CV. C) Examples of test for non-specific binding of patient serum demonstrating IgG reactivity to the BMPR2 ECD. Samples were incubated both in the presence and absence of ECD and calculated as percentage binding. Sera with high levels of non-specific absorbance were removed.

179x133mm (300 x 300 DPI)

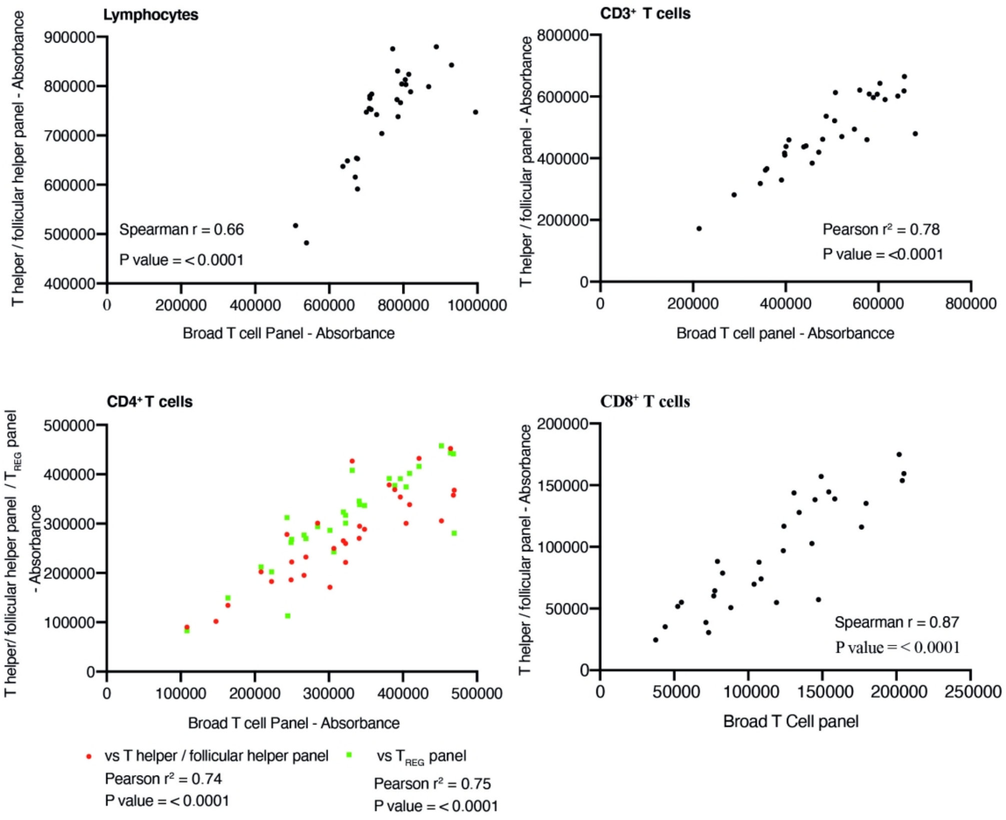

Supplementary Figure E4: Correlation analysis of lymphocytes; CD3+; CD4+ T cell and CD8+ T cell populations measured across different immunophenotyping panels. Samples from individuals were measured across multiple antibody panels and show good correlation. P values and r or r2 were derived by Pearson or Spearman’s rank correlation determined by normality of data.

205x168mm (300 x 300 DPI)

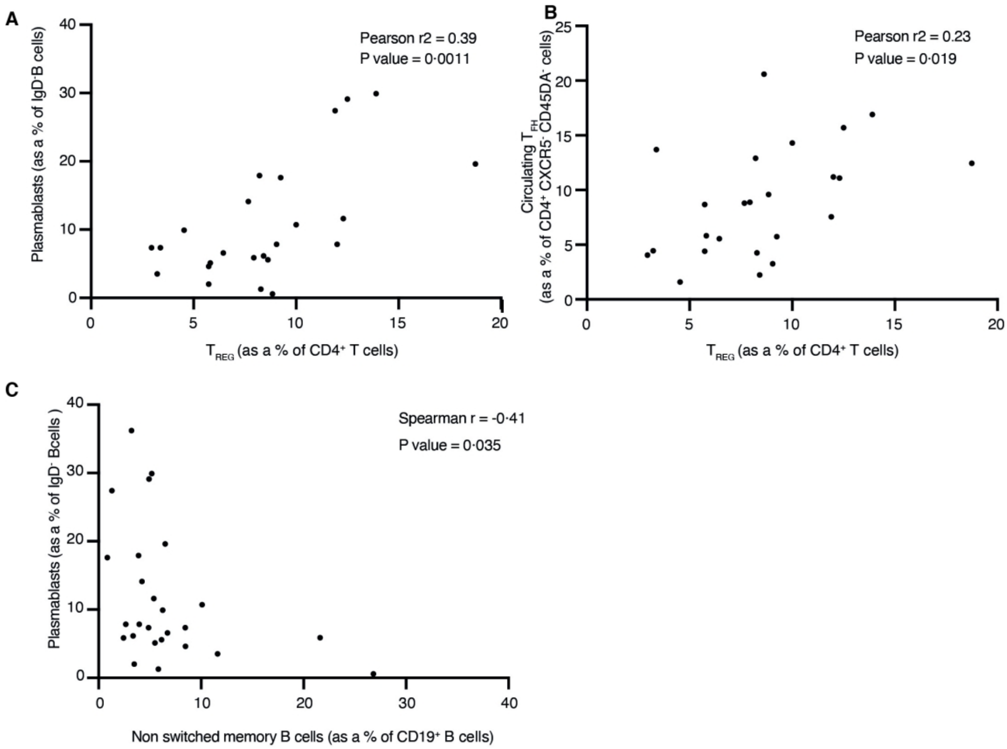

Supplementary Figure E5: Correlations of leukocyte cell populations in IPAH patients. A) TREG vs. Plasmablast; B) TREG vs. circulating TFH; C) Non-switched memory B cells vs. Plasmablasts. P values and r calculations derived from Pearson or Spearmans rank correlation depending on normality of data.

185x138mm (300 x 300 DPI)

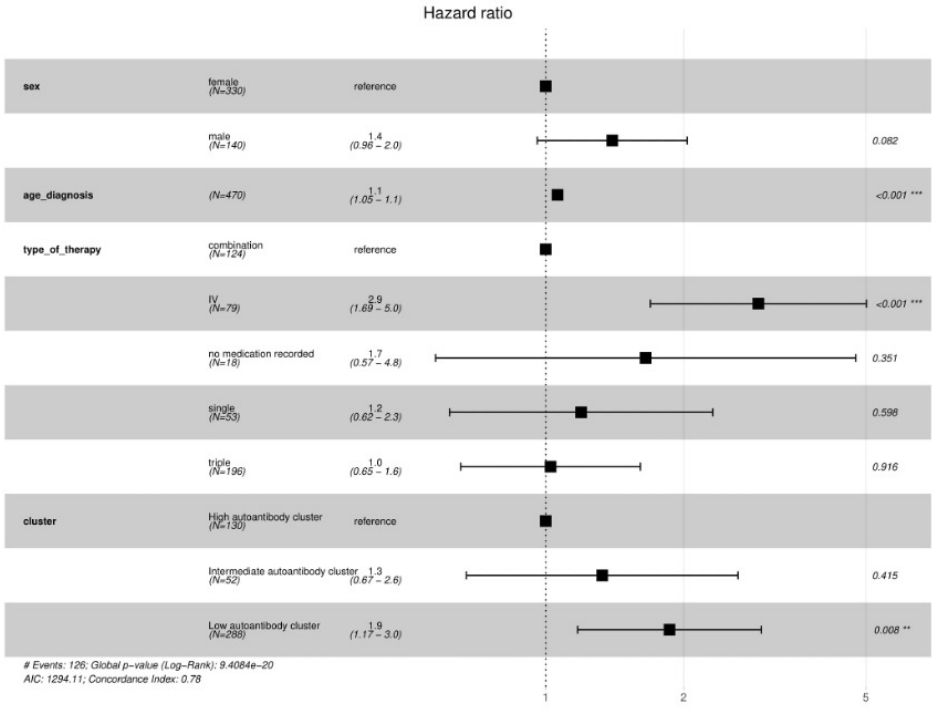

Supplementary Figure E6: Cox-proportional hazard model of survival differences between clusters. Survival differences between clusters were corrected for age at diagnosis, sex and treatment using a cox-proportional hazard model. Wald tests were used to determine p-values.

159x115mm (144 x 144 DPI)
